# Associations of cholinergic system integrity with cognitive decline in GBA1 and LRRK2 mutation carriers

**DOI:** 10.1038/s41531-024-00743-w

**Published:** 2024-06-29

**Authors:** Julia Schumacher, Nicola Ray, Stefan Teipel, Alexander Storch

**Affiliations:** 1 Department of Neurology, University Medical Center Rostock, 18147 Rostock, Germany; 2 Deutsches Zentrum für Neurodegenerative Erkrankungen (DZNE) Rostock-Greifswald, 18147 Rostock, Germany; 3 Health, Psychology and Communities Research Centre, Department of Psychology, Manchester Metropolitan University, Manchester, UK; 4 Department of Psychosomatic Medicine, University Medical Center Rostock, 18147 Rostock, Germany

**Keywords:** Parkinson's disease, Prognostic markers

## Abstract

In Parkinson’s disease (PD), GBA1- and LRRK2-mutations are associated with different clinical phenotypes which might be related to differential involvement of the cholinergic system. We investigated cholinergic integrity in 149 asymptomatic GBA1 and 169 asymptomatic LRRK2 mutation carriers, 112 LRRK2 and 60 GBA1 carriers with PD, 492 idiopathic PD, and 180 controls from the PPMI cohort. Basal forebrain volumes were extracted and white matter pathways from nucleus basalis of Meynert (NBM) to cortex and from pedunculopontine nucleus (PPN) to thalamus were assessed with a free water-corrected DTI model. Bayesian ANCOVAs were conducted for group comparisons and Bayesian linear mixed models to assess associations with cognitive decline. Basal forebrain volumes were increased in asymptomatic GBA1 (Bayes Factor against the null hypothesis (BF_10_) = 75.2) and asymptomatic LRRK2 (BF_10_ = 57.0) compared to controls. Basal forebrain volumes were increased in LRRK2- compared to GBA1-PD (BF_10_ = 14.5) and idiopathic PD (BF_10_ = 3.6*10^7^), with no difference between idiopathic PD and PD-GBA1 (BF_10_ = 0.25). Mean diffusivity along the medial NBM pathway was decreased in asymptomatic GBA1 compared to controls (BF_10_ = 30.3). Over 5 years, idiopathic PD and PD-GBA1 declined across all cognitive domains whereas PD-LRRK2 patients only declined in processing speed. We found an interaction between basal forebrain volume and time in predicting multiple cognitive domains in idiopathic PD and PD-GBA1, but not in PD-LRRK2. While LRRK2 and GBA1 mutations are both associated with increased basal forebrain volume at asymptomatic stages, this increase persists at the symptomatic PD stage only in LRRK2 and might be related to slower cognitive decline in these patients.

## Introduction

Mutations in the genes encoding for glucocerebrosidase (GBA1) and leucine-rich repeat kinase 2 (LRRK2) are among the most common genetic risk factors for Parkinson’s disease (PD)^1^. Generally, mutations in GBA1 are associated with a more severe clinical progression, including earlier age of onset, earlier and more severe cognitive impairment with a shorter time to dementia and higher frequency of neuropsychiatric symptoms compared to idiopathic PD^2–7^. In contrast, PD patients with LRRK2 mutations generally show a more motor-predominant disease course with less cognitive decline and slower disease progression^8–10^.

An important driver of cognitive impairment in PD are changes within the cholinergic system^11–14^. There are two major sources of cholinergic projections in the brain. First, the nucleus basalis of Meynert (NBM) in the basal forebrain provides the main source of cortical cholinergic input via its widespread connections to the entire cortex^15^. Second, the pedunculopontine nucleus (PPN) located in the brainstem provides cholinergic innervation primarily to the thalamus^16,17^, thereby influencing thalamocortical activity^18^.

In PD, degeneration of cholinergic neurons in the basal forebrain is associated with the severity of cognitive impairment and has been shown to be predictive of future cognitive decline^11–14^. In people with LRRK2 mutations, there is evidence from a PET study of an increase in cortical cholinergic activity, already found in asymptomatic mutation carriers, which has been suggested to be a compensatory mechanism associated with the relatively preserved cognitive function in these patients^19,20^. In line with this, a recent small-sized study found an increase in basal forebrain volumes in asymptomatic LRRK2 mutation carriers (*N* = 13) and PD patients with LRRK2 mutations (*N* = 31)^21^. Evidence of cholinergic changes in GBA1 mutation carriers is restricted to one cholinergic PET study, reporting more widespread cortical cholinergic deficits in PD-GBA1 compared to idiopathic PD when both groups are compared to healthy controls^22^. However, we do not know if cholinergic integrity is altered in asymptomatic GBA1 mutation carriers, or whether any loss of cholinergic function in this group is associated with cognitive health. It is also not known whether the cholinergic deficits observed in LRRK2 mutation carriers are different to those reported for GBA1 mutation carriers.

The present study therefore aimed to conduct a comprehensive investigation of the basal forebrain and PPN cholinergic projection systems in GBA1 and LRRK2 mutation carriers with and without manifest PD compared to idiopathic PD and similarly aged healthy controls. The second aim was to better understand the relationship between cholinergic changes and the development of cognitive impairment and how this might differ between the genetic groups. For the basal forebrain, we conducted a volumetric analysis and investigated the two main cortically-projecting white matter pathways described in previous studies using a free-water-corrected diffusion-weighted imaging (DWI) analysis^23,24^. For the PPN, due to its brainstem location, a volumetric analysis is less meaningful. We therefore focussed on diffusion metrics in the PPN itself as well as the integrity of its white matter pathways projecting to the thalamus^25^.

Based on previous evidence and the differential cognitive symptom profile we hypothesised that we would find a more intact cholinergic system in LRRK2 mutation carriers compared to idiopathic PD. We also expected to see similar or more severe cholinergic degeneration in PD-GBA1 compared to non-mutation carriers with PD. Due to the lack of a direct comparison of the cholinergic system between GBA1 and LRRK2 mutation carriers in previous studies, we did not have specific hypotheses for analyses that will compare the two genetic groups, or the nature of any differences in relationships between cholinergic integrity and longitudinal cognitive changes between the two groups.

## Results

### Demographics

Demographic and clinical information for the six groups can be found in Table 1 (and in Supplementary Table 1 for the subset of participants included in the free-water DWI analysis). Asymptomatic carriers and healthy controls were comparable in age, as were the three PD groups. The ratio of male and female participants was similar in the three PD groups whereas sex was unequally distributed in the asymptomatic mutation carriers compared to controls. The asymptomatic mutation carriers already showed slight changes in overall cognition and UPDRS motor scores. Disease duration was longer in the PD mutation groups compared to idiopathic PD due to the difference in inclusion criteria for these groups and was therefore included as an additional covariate in the models for PD group comparisons.

**Table 1:** Demographic and clinical information

	**Control (*N*** = 180)	**Asymptomatic GBA1 (*N*** = 169)	**Asymptomatic LRRK2 (*N*** = 149)	**Group comparison**
Age at baseline	62.8 (8.2)	61.8 (6.7)	61.1 (8.2)	BF_10_ = 0.16^a^
Male:female	116:64	66:103	60:89	BF_10_ = 29765^b^
Years of education	16.2 (3.0)	18.0 (2.6)	16.8 (3.7)	BF_10_ = 20054^a^, GBA1 > LRRK2, HC
MoCA	28.0 (1.4)	26.8 (2.2)	27.1 (2.2)	BF_10_ = 650956, HC > GBA1, LRRK2^a^
UPDRS III (OFF)	1.1 (1.8)	2.6 (3.9)	2.6 (3.9)	BF_10_ = 1199, HC < GBA1, LRRK2^a^
Aβ_1-42_ (pg/ml)	1052.6 (517.8)^c^	939.2 (459.6)^d^	1120.0 (492.2)^e^	BF_10_ = 0.15^a^
P-tau_181_ (pg/ml)	17.4 (9.0)^f^	14.6 (5.4)^g^	15.1 (5.7)^h^	BF_10_ = 9.5^a^, HC > GBA1
Total tau (pg/ml)	195.0 (82.8)^f^	171.3 (64.2)^g^	175.2 (63.5)^h^	BF_10_ = 1.7^a^
Positive DATscan, *N* (%)	–	10 (6)	11 (17)	
*N* with follow-up data				
Year 1	–	155	139	
Year 2	–	133	121	
Year 3	–	127	105	
Year 4	–	119	109	
Year 5	–	92	87	
	**Idiopathic PD (*N*** = 492)	**PD GBA1 (*N*** = 60)	**PD LRRK2 (*N*** = 112)	**Group comparison**
Age at baseline	62.3 (9.7)	61.4 (11.0)	63.7 (9.0)	BF_10_ = 0.098^a^
Male:female	318:174	34:26	61:51	BF_10_ = 0.097^b^
Years of education	16.0 (3.2)	16.6 (3.3)	15.5 (4.6)	BF_10_ = 0.20^a^
MoCA	27.0 (2.4)	26.5 (2.6)	26.0 (3.1)	BF_10_ = 51.5^a^, LRRK2 < iPD
UPDRS III (OFF)	21.5 (10.4)	28.0 (10.9)	22.0 (10.6)	BF_10_ = 261.4, GBA1 > iPD, LRRK2^a^
Disease duration (in years)	0.69 (0.71)	2.36 (1.99)	2.87 (2.06)	BF_10_ = 1.6 × 10^60^, iPD < GBA1, LRRK2^a^
Aβ_1-42_ (pg/ml)	910.5 (409.3)^i^	896.3 (393.4)^j^	879.6 (398.0)^k^	BF_10_ = 0.08^a^
P-tau_181P_ (pg/ml)	14.3 (5.5)^l^	14.4 (6.8)^m^	14.5 (5.6)^n^	BF_10_ = 0.04^a^
Total tau (pg/ml)	167.2 (59.1)^l^	166.1 (73.5)^m^	169.6 (64.2)^n^	BF_10_ = 0.04^a^
*N* with follow-up data				
Year 1	406	52	102	
Year 2	306	45	88	
Year 3	285	37	82	
Year 4	269	35	80	
Year 5	240	30	64	

Mean (standard deviation).

*Aβ*_*1-42*_ CSF amyloid-β 1-42, *BF*_*10*_ Bayes factor quantifying evidence against the null hypothesis, *iPD* idiopathic Parkinson’s disease, *MoCA* Montreal Cognitive Assessment, *ptau*_*181*_ CSF phosphorylated tau 181, *UPDRS* Unified Parkinson’s disease rating scale.

^a^Bayesian ANOVA all groups (with post hoc tests).

^b^Bayesian contingency tables test.

^c^*N* = 139.

^d^*N* = 7.

^e^*N* = 36.

^f^*N* = 141.

^g^*N* = 142.

^h^*N* = 123.

^i^*N* = 314.

^j^*N* = 14.

^k^*N* = 53.

^l^*N* = 317.

^m^*N* = 42.

^n^*N* = 100.

Ten of the asymptomatic GBA1 and 21 of the asymptomatic LRRK2 participants had a positive DATscan.

There were no differences between the groups in CSF biomarkers of Alzheimer’s disease pathology, except for higher levels of phosphorylated tau_181_ in controls compared to asymptomatic GBA1 carriers (Table 1).

Over a median follow-up time of 5 years (range: 0–9 years), three of the asymptomatic LRRK2 carriers and one asymptomatic GBA1 carrier phenoconverted to PD.

### Group comparison of volumes

**Fig. 1:**
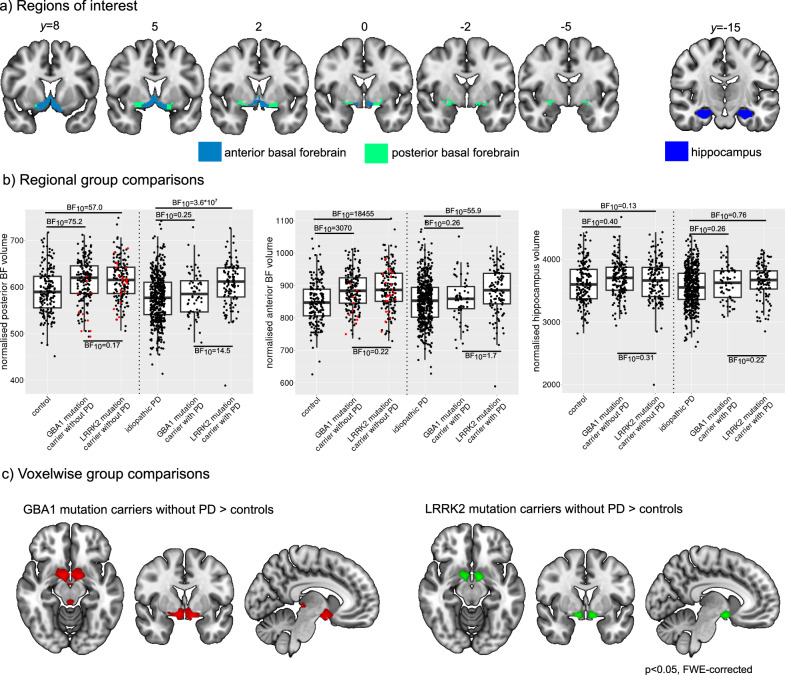
Volumetric group differences. **a** Masks that were used for the extraction of volumes from the anterior and posterior basal forebrain and the hippocampus. **b** Group comparison of subregional basal forebrain and hippocampus volume (normalised with respect to total intracranial volume). In each box plot the central line corresponds to the sample median, the upper and lower border of the box represent the 25th and 75th percentile, respectively, and the length of the whiskers corresponds to 1.5x the interquartile range. Bayes factors quantifying evidence against the null hypothesis (BF_10_) from Bayesian ANCOVAs including covariates for age, sex, years of education (and disease duration for the PD groups). Statistical results for all comparisons can be found in Table 2. Participants in the asymptomatic cohorts with a positive DATscan result are marked in red. **c** Significant clusters (FWE-corrected *p* < 0.05) from whole-brain voxelwise comparison of volumes between asymptomatic mutation carriers and healthy controls. BF basal forebrain, FWE family-wise error, PD Parkinson’s disease.

We found evidence for an increase in posterior and anterior basal forebrain volume in both asymptomatic mutation carrier groups compared to healthy controls (Table 2, Fig. 1b and Supplementary Fig. 1). When comparing the two asymptomatic mutation carrier groups, there was evidence in favour of the null hypothesis, i.e. no difference. At the PD stage, posterior basal forebrain volume was increased in PD-LRRK2 compared to idiopathic PD and PD-GBA1 whereas there was evidence for no difference between idiopathic PD and PD-GBA1. Anterior basal forebrain volume was increased in PD-LRRK2 compared to idiopathic PD, not different between idiopathic PD and PD-GBA1 and the evidence for the comparison of the two mutation groups was inconclusive. When restricting data to those from 3T Siemens scanners, the results at the asymptomatic stage persisted (Supplementary Table 3 and Supplementary Figs. 2–4). At the PD stage, LRRK2 mutation carriers showed increased posterior and anterior basal forebrain volume compared to idiopathic PD, consistent with the analysis of the full cohort. The results with respect to the PD-GBA1 group changed slightly with no evidence for differences in basal forebrain volume between PD-GBA1 and PD-LRRK2 and moderate evidence for an increase in anterior basal forebrain volume in PD-GBA1 compared to idiopathic PD.

**Table 2:** Group comparisons of basal forebrain and hippocampus volume (normalised with respect to total intracranial volume) by Bayesian ANCOVAs, including covariates for age, sex, years of education, and disease duration (only for PD groups)

	Posterior basal forebrain volume	Anterior basal forebrain volume	Hippocampus volume
HC vs. asympt. GBA1	**BF**_**10**_ = **75.2**	**BF**_**10**_ = **3070**	BF_10_ = 0.40
HC vs. asympt. LRRK	**BF**_**10**_ = **57.0**	**BF**_**10**_ = **18455**	BF_10_ = 0.13
Asympt. GBA1 vs. asympt. LRRK2	BF_10_ = 0.17	BF_10_ = 0.22	BF_10_ = 0.31
iPD vs. PD-GBA1	BF_10_ = 0.25	BF_10_ = 0.26	BF_10_ = 0.26
iPD vs. PD-LRRK2	**BF**_**10**_ = **3.6** **×** **10**^**7**^	**BF**_**10**_ = **55.9**	BF_10_ = 0.76
PD-GBA1 vs. PD-LRRK2	**BF**_**10**_ = **14.5**	BF_10_ = 1.7	BF_10_ = 0.22
HC vs. iPD	**BF**_**10**_ = **236.0**	BF_10_ = 0.11	BF_10_ = 2.69
HC vs. PD-GBA1	BF_10_ = 0.44	BF_10_ = 0.19	BF_10_ = 0.19
HC vs. PD-LRRK2	BF_10_ = 2.65	**BF**_**10**_ = **414.7**	BF_10_ = 0.24
Asympt. GBA1 vs. PD-GBA1	**BF**_**10**_ = **455.0**	**BF**_**10**_ = **5.24**	BF_10_ = 0.68
Asympt. LRRK2 vs. PD-LRRK2	BF_10_ = 0.23	BF_10_ = 0.18	BF_10_ = 0.16

*BF* basal forebrain, *BF*_*10*_ Bayes factor quantifying evidence against the null hypothesis, *HC* healthy controls, *iPD* idiopathic Parkinson’s disease.

Bayes factors (BF_10_)>3 are marked in bold.

The comparison between PD groups and healthy controls revealed decreased posterior basal forebrain volume in idiopathic PD and increased anterior basal forebrain volume in PD-LRRK2. When comparing asymptomatic mutation carriers to carriers of the same mutation at the PD stage, we found a decrease in posterior and anterior basal forebrain volume in PD-GBA1 compared to asymptomatic GBA1 carriers whereas for LRRK2 we found evidence for no difference between asymptomatic mutation carriers and mutation carriers with PD (Table 2). There was no evidence for differences in hippocampus volume between any of the groups (Fig. 1b, Table 2 and Fig. S1), which remained in the analysis of 3T Siemens data (Supplementary Table 3 and Supplementary Figs. 2 and 3).

Figure 1c shows clusters from whole-brain voxelwise group comparisons (restricted to 3T Siemens data). For the asymptomatic GBA1 > controls and asymptomatic LRRK2 > controls comparisons, significant clusters (FWE-corrected *p* < 0.05) were mainly found in the region of the basal forebrain, with an additional cluster in the thalamus for the asymptomatic GBA1 > controls comparison. At the PD stage, we found no significant clusters for any comparison, except for a small cluster (20 voxels) of increased volume in PD-LRRK2 compared to idiopathic PD in the right brainstem.

In Fig. 1b, the asymptomatic mutation carriers with a positive DATscan are marked in red. Bayesian ANCOVAs revealed that posterior (BF_10_ = 3126) and anterior basal forebrain volume (BF_10_ = 12.7) were smaller in GBA1 mutation carriers with a positive compared to those with a negative DATscan. In the asymptomatic LRRK2 group, posterior (BF_10_ = 0.34) and anterior basal forebrain volume (BF_10_ = 0.28) were not different between the two subgroups.

When restricting the analysis to only include mutation carriers with the most common mutations (i.e. G2019S for LRRK2 and N409S for GBA1), the results were comparable to the analysis of the whole cohort (Supplementary Table 3).

We did not find evidence for differences in basal forebrain or hippocampus volume between those PD-GBA1 or PD-LRRK2 patients who were and those who were not taking PD-related medications (Supplementary Table 4).

Supplementary Table 7 includes results from a standard frequentist statistical approach (ANCOVAs with post hoc tests corrected for multiple comparisons), indicating that in most cases a BF_10_ value of about 10 or higher would be required for statistical significance as defined in the frequentist framework (i.e. corresponding to *p* value < 0.05).

### Group comparison of free-water DTI metrics

**Fig. 2:**
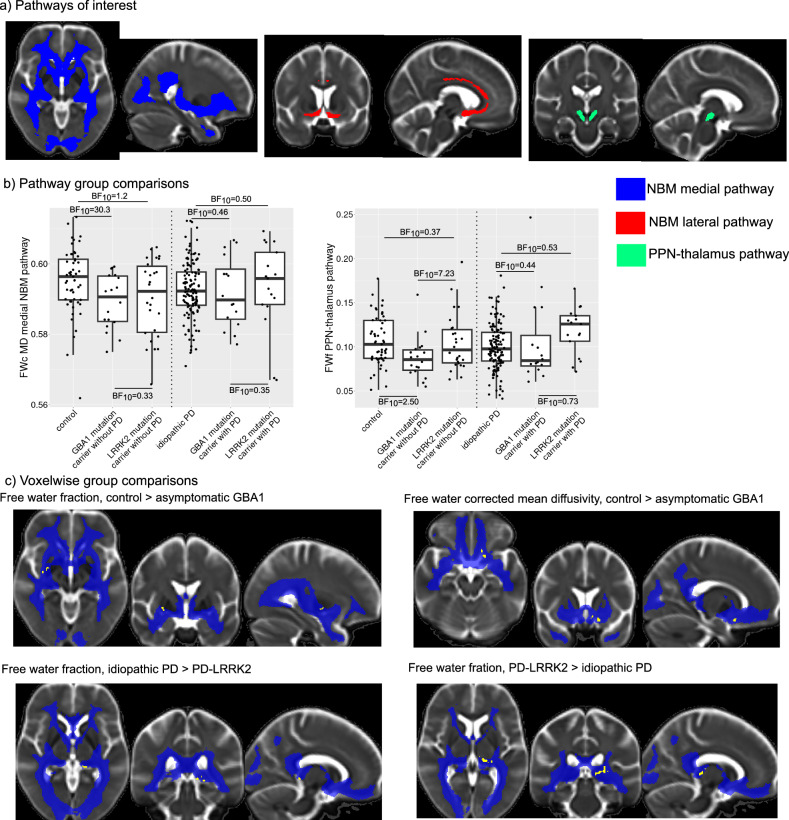
Group differences in free water DTI metrics. **a** Estimated cholinergic white matter pathways that were used for the extraction of free water DTI metrics. **b** Group comparison of free water DTI metrics from the medial NBM and PPN-thalamus pathway that showed some evidence in favour of the alternative hypothesis. Bayes factors quantifying evidence against the null hypothesis (BF_10_) from Bayesian ANCOVAs including covariates for age, sex, years of education (and disease duration for the PD groups). Statistical results for all comparisons can be found in Tables 3 and 4. In each box plot the central line corresponds to the sample median, the upper and lower border of the box represent the 25th and 75th percentile, respectively, and the length of the whiskers corresponds to 1.5x the interquartile range. **c** Significant clusters (*p*_TFCE-corrected_ < 0.05, shown in yellow) from group comparisons of DTI metrics for the lateral NBM pathway (shown in blue). Here only clusters with a minimum size of five voxels are shown. A list of clusters for all comparisons can be found in Supplementary Table 3.

Figure 2b and Tables 3 and 4 show group comparisons of the free-water DTI metrics (posterior distributions for parameter estimates in Supplementary Figs. 5 and 6). For most comparisons, the Bayes factor either indicated moderate evidence for the null hypothesis (0.1 < BF_10_ < 0.33) or was in the inconclusive range (0.33 < BF_10_ < 3). The only comparisons with moderate evidence for the alternative hypothesis were a decrease in free water-corrected mean diffusivity (cMD) in asymptomatic GBA1 mutation carriers compared to controls along the medial pathway, and reduced free water fraction in asymptomatic GBA1 compared to asymptomatic LRRK2 mutation carriers in the PPN-thalamus pathway. To test the robustness of the results with respect to the relatively small sample size of the DWI subsample, we also conducted a Bayesian sequential analysis in which the Bayes Factor is evaluated after sequentially adding one observation at a time (see Supplementary Figs. 7 and 8).

**Table 3:** Group comparisons of free water fraction and free water-corrected diffusivity metrics in the NBM pathways by Bayesian ANCOVAs, including covariates for age, sex, years of education, diffusivity metric from the respective control mask and disease duration (for PD groups)

	Free water fraction		Free water corrected mean diffusivity		Free water corrected axial diffusivity	
	NBM lateral tract	NBM medial tract	NBM lateral tract	NBM medial tract	NBM lateral tract	NBM medial tract
HC vs. asympt. GBA1	BF_10_ = 0.31	BF_10_ = 0.32	BF_10_ = 0.31	**BF**_**10**_ = **30.3**	BF_10_ = 0.31	BF_10_ = 0.77
HC vs. asympt. LRRK	BF_10_ = 0.88	BF_10_ = 0.49	BF_10_ = 2.97	BF_10_ = 1.18	BF_10_ = 0.49	BF_10_ = 0.34
Asympt. GBA1 vs. asympt. LRRK2	BF_10_ = 0.40	BF_10_ = 0.41	BF_10_ = 0.71	BF_10_ = 0.33	BF_10_ = 0.31	BF_10_ = 0.38
iPD vs. PD-GBA1	BF_10_ = 0.37	BF_10_ = 0.28	BF_10_ = 0.40	BF_10_ = 0.46	BF_10_ = 0.30	BF_10_ = 0.30
iPD vs. PD-LRRK2	BF_10_ = 0.30	BF_10_ = 0.27	BF_10_ = 0.52	BF_10_ = 0.50	BF_10_ = 0.36	BF_10_ = 0.31
PD-GBA1 vs. PD-LRRK2	BF_10_ = 0.37	BF_10_ = 0.35	BF_10_ = 0.59	BF_10_ = 0.35	BF_10_ = 0.83	BF_10_ = 0.36
HC vs. iPD	BF_10_ = 0.98	BF_10_ = 0.26	BF_10_ = 0.69	BF_10_ = 0.18	BF_10_ = 0.19	BF_10_ = 0.17
HC vs. PD-GBA1	BF_10_ = 0.56	BF_10_ = 0.58	BF_10_ = 0.29	BF_10_ = 0.41	BF_10_ = 0.35	BF_10_ = 0.29
HC vs. PD-LRRK2	BF_10_ = 0.39	BF_10_ = 0.44	BF_10_ = 0.37	BF_10_ = 0.33	BF_10_ = 0.37	BF_10_ = 0.36
Asympt. GBA1 vs. PD-GBA1	BF_10_ = 1.28	BF_10_ = 0.60	BF_10_ = 0.39	BF_10_ = 0.41	BF_10_ = 0.52	BF_10_ = 1.75
Asympt. LRRK2 vs. PD-LRRK2	BF_10_ = 0.37	BF_10_ = 0.35	BF_10_ = 0.48	BF_10_ = 0.32	BF_10_ = 1.10	BF_10_ = 0.34

*BF*_*10*_ Bayes factor quantifying evidence against the null hypothesis, *HC* healthy controls, *iPD* idiopathic Parkinson’s disease, *NBM* nucleus basalis of Meynert.

Bayes factors (BF_10_)>3 are marked in bold.

**Table 4:** Group comparisons of free water fraction and free water-corrected diffusivity metrics in the PPN and PPN-thalamus pathway by Bayesian ANCOVAs, including covariates for age, sex, years of education, diffusivity metric from the respective control mask and disease duration (for PD groups)

	Free water fraction		Free water corrected mean diffusivity		Free water corrected axial diffusivity	
	PPN	PPN thalamus tract	PPN	PPN thalamus tract	PPN	PPN thalamus tract
HC vs. asympt. GBA1	BF_10_ = 0.50	BF_10_ = 2.50	BF_10_ = 0.29	BF_10_ = 0.38	BF_10_ = 0.33	BF_10_ = 2.86
HC vs. asympt. LRRK	BF_10_ = 0.34	BF_10_ = 0.37	BF_10_ = 0.32	BF_10_ = 0.80	BF_10_ = 0.32	BF_10_ = 0.40
Asympt. GBA1 vs. asympt. LRRK2	BF_10_ = 0.45	**BF**_**10**_ = **7.23**	BF_10_ = 0.44	BF_10_ = 0.40	BF_10_ = 0.93	BF_10_ = 0.69
iPD vs. PD-GBA1	BF_10_ = 0.92	BF_10_ = 0.44	BF_10_ = 0.29	BF_10_ = 0.23	BF_10_ = 0.28	BF_10_ = 0.23
iPD vs. PD-LRRK2	BF_10_ = 0.31	BF_10_ = 0.53	BF_10_ = 0.33	BF_10_ = 0.34	BF_10_ = 0.31	BF_10_ = 0.31
PD-GBA1 vs. PD-LRRK2	BF_10_ = 0.60	BF_10_ = 0.73	BF_10_ = 0.49	BF_10_ = 1.61	BF_10_ = 0.33	BF_10_ = 0.39
HC vs. iPD	BF_10_ = 0.36	BF_10_ = 0.22	BF_10_ = 0.32	BF_10_ = 0.21	BF_10_ = 0.44	BF_10_ = 0.48
HC vs. PD-GBA1	BF_10_ = 0.53	BF_10_ = 0.48	BF_10_ = 0.33	BF_10_ = 0.36	BF_10_ = 0.39	BF_10_ = 0.31
HC vs. PD-LRRK2	BF_10_ = 0.32	BF_10_ = 0.63	BF_10_ = 0.52	BF_10_ = 0.32	BF_10_ = 0.59	BF_10_ = 0.45
Asympt. GBA1 vs. PD-GBA1	BF_10_ = 1.40	BF_10_ = 1.79	BF_10_ = 0.39	BF_10_ = 0.70	BF_10_ = 0.62	BF_10_ = 0.59
Asympt. LRRK2 vs. PD-LRRK2	BF_10_ = 0.43	BF_10_ = 0.39	BF_10_ = 0.56	BF_10_ = 0.47	BF_10_ = 0.94	BF_10_ = 0.67

*BF*_*10*_ Bayes factor quantifying evidence against the null hypothesis, *HC* healthy controls, *iPD* idiopathic Parkinson’s disease, *NBM* nucleus basalis of Meynert.

Bayes factors (BF_10_)>3 are marked in bold.

Supplementary Table 5 shows all clusters with TFCE-corrected *p* < 0.05 from the voxelwise analysis of DTI metrics along the estimated tracts. Clusters with more than five voxels are shown in Fig. 2c, indicating some small clusters of group differences along the medial NBM pathway.

We did not find any evidence for differences in any diffusivity metrics between those PD-GBA1 or PD-LRRK2 patients who were and those who were not taking PD-related medications (Supplementary Table 4).

### Longitudinal changes in cognition

**Fig. 3:**
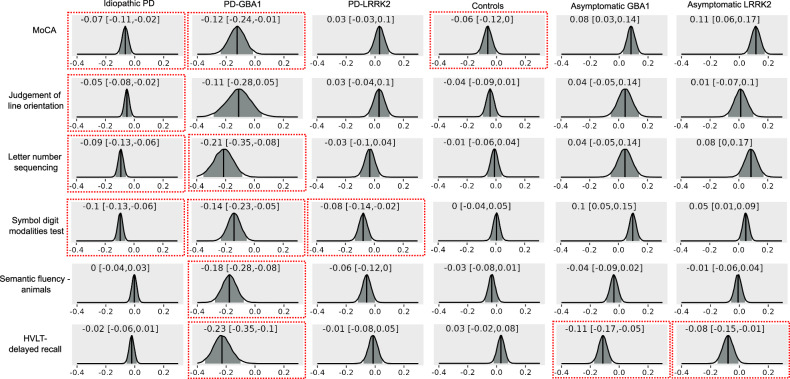
Longitudinal changes in cognition. Posterior distributions of parameter estimates for the effect of time in Bayesian mixed models including cognitive scores as dependent variable, fixed effects for time, age, sex, years of education (and disease duration for PD groups), and random effects for intercepts and time. The median is marked with a solid line and the 95% credible intervals in grey, and stated in each plot. Parameter estimates for which the 95% credible interval does not overlap with zero are marked with a red box. HVLT Hopkins Verbal Learning Test, MoCA Montreal Cognitive Assessment, PD Parkinson’s disease.

Supplementary Table 6 shows cognitive scores over time in the different diagnostic groups. Figure 3 shows posterior distributions of the parameter estimates of the effect of time on cognitive scores in Bayesian mixed models including covariates for age, sex, years of education (and disease duration in the PD groups). Idiopathic PD and PD-GBA1 groups showed cognitive decline in multiple cognitive domains including global cognition, visuospatial function, executive function, processing speed, and memory, whereas in the PD-LRRK2 patients cognitive decline was only evident for processing speed. The asymptomatic GBA1 and LRRK2 mutation carriers showed decline in episodic memory.

### Effect of basal forebrain volume on changes in cognition

**Fig. 4:**
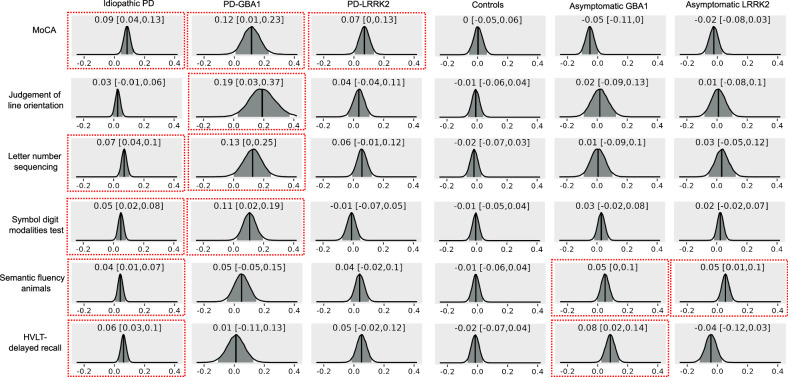
Association between posterior basal forebrain volume and changes in cognition. Posterior distributions of parameter estimates for the interaction of time and posterior basal forebrain volume in Bayesian mixed models including cognitive scores as dependent variable, fixed effects for posterior basal forebrain volume, time, age, sex, years of education (and disease duration for PD groups), the interaction between time and posterior basal forebrain volume, and random effects for intercepts and time. The median is marked with a solid line and the 95% credible intervals in grey, and stated in each plot. Parameter estimates for which the 95% credible interval does not overlap with zero are marked with a red box. HVLT Hopkins Verbal Learning Test, MoCA Montreal Cognitive Assessment, PD Parkinson’s disease.

Posterior distributions of the parameter estimates of the interaction between time and posterior basal forebrain volume in predicting cognitive scores are shown in Fig. 4. In idiopathic PD, baseline posterior basal forebrain volumes were related to changes in all cognitive domains, except for visuospatial function. There were similar associations with anterior basal forebrain volume except for semantic fluency (Supplementary Fig. 9). In PD-GBA1, posterior basal forebrain volume predicted changes in multiple cognitive domains, including global cognition, visuospatial function, executive function, and processing speed whereas there were no associations with anterior basal forebrain volume. In the PD-LRRK2 patients only global cognitive changes were related to posterior and anterior basal forebrain volume. In both asymptomatic mutation carrier groups there was an association with semantic fluency and additionally with episodic memory scores in the asymptomatic GBA1 participants.

## Discussion

We found larger basal forebrain volumes in asymptomatic GBA1 mutation carriers compared to healthy controls, which was accompanied by more intact cholinergic pathways in the asymptomatic GBA1 group. At the PD stage, basal forebrain volumes and cholinergic pathway integrity were similar in the GBA1 mutation carriers compared to idiopathic PD. These findings imply that basal forebrain volumes degenerate in GBA1 carriers as PD progresses, potentially at a faster rate than is seen in idiopathic PD. The analysis of cholinergic pathways with DTI showed changes mostly consistent with the basal forebrain volumetric analysis in that asymptomatic GBA1 mutation carriers had more intact structural metrics in cholinergic basal forebrain pathways than healthy controls. The reduction in free water fraction and free water-corrected diffusivity measures indicates more intact structure of both basal forebrain and PPN-originating cholinergic pathways in asymptomatic people with GBA1 mutations compared to healthy non-mutation carriers, which may become lost at the PD stage.

These findings may seem inconsistent with a previous cholinergic PET study in PD-GBA1 patients, which found more severe cholinergic deficits in PD-GBA1 than in idiopathic PD when compared to controls^22^. The PD-GBA1 group in Slingerland et al. is comparable to the current sample in terms of disease duration, MOCA, and UPDRS motor scores. The discrepancy in findings may therefore indicate that cortical cholinergic changes in PD-GBA1 might be detectable earlier than basal forebrain volumetric changes, similar to what has been reported in early dementia with Lewy bodies^26^. It should also be noted that in Slingerland et al. the direct comparison between the idiopathic PD and PD-GBA1 groups did not yield significant results and the cortical cholinergic maps showed high overlap between both PD groups, indicating that effect sizes are modest^22^.

In addition, there may be complex relationships between cholinergic activity measured with PET and both volumetric and diffusivity-based structural markers of cholinergic system integrity, with synaptic upregulation likely occurring in early and prodromal disease stages alongside structural degeneration. It is not known, however, if these relationships differ in idiopathic compared to genetic PD. An analysis of different cholinergic markers in the same patients as well as a cholinergic PET study in asymptomatic GBA1 carriers will help reconcile these findings.

We also observed larger basal forebrain volumes in asymptomatic LRRK2 mutation carriers compared to healthy controls, which was still evident in those who had progressed to PD diagnosis. These findings are consistent with a recent investigation of basal forebrain volumes^21^ and a previous cholinergic PET study reporting a hypercholinergic state associated with LRRK2 mutations in asymptomatic individuals as well as those with a PD diagnosis^19^. As such, unlike in GBA1 mutation carriers, LRRK2 mutations may be associated with preserved, or even superior cholinergic integrity compared with controls and people with PD with and without GBA1 mutations, at least throughout early disease stages. The latter suggestion is consistent with our finding of no differences between LRRK2 mutation carriers with PD and those who were still asymptomatic.

In contrast to the volumetric analysis, we did not find differences in the LRRK2 groups compared to controls or idiopathic PD patients for the analysis of tract integrity. The sequential analysis showed that the Bayes Factors did not always converge into one direction (fluctuating around a Bayes Factor of 1), indicating that this might also be related to the much smaller sample size of the DWI analysis (see “Limitations” section).

The analysis of hippocampal volume illustrates the specificity of our volumetric findings for the cholinergic system and is consistent with previous research showing that cholinergic changes are a more important driver of early cognitive impairment in PD than hippocampal atrophy^27–29^. At the asymptomatic stage, the specificity of volumetric changes to the cholinergic system is further underlined by the whole-brain voxelwise grey matter analysis which identified clusters of increased volume compared to healthy controls mainly restricted to the region of the basal forebrain.

Over the course of 5 years, idiopathic PD and PD-GBA1 patients declined across all cognitive domains whereas the PD-LRRK2 group only declined in processing speed which is in line with previous findings on cognitive symptom progression in these groups^6,21,30^. We found that more severe decline in multiple cognitive domains was related to smaller posterior and anterior basal forebrain volumes at baseline in idiopathic PD and to smaller posterior basal forebrain volumes in PD-GBA1. In idiopathic PD, this association between cholinergic degeneration and future cognitive decline is a well-established finding^11,12,14^. Our results suggest that a similar relationship exists in PD-GBA1, consistent with the similar cognitive symptom profile in these patients. In the PD-LRRK2 group, there was no association of posterior or anterior basal forebrain volumes with longitudinal measures of domain-specific cognition, consistent with the hypothesis that there is a relationship between preserved cholinergic function and the absence of cognitive decline in LRRK2 mutation carriers^21^.

GBA1 and LRRK2 asymptomatic mutation carriers showed longitudinal decline in verbal memory scores which has been reported previously in a small group of non-manifesting GBA1 mutation carriers^31^. The identified association with baseline posterior basal forebrain volumes in our study suggests that memory decline is more severe in those GBA1 mutation carriers that show early cholinergic degeneration.

Overall, the results of the present study in addition to previous work point toward a more intact cholinergic system in people with mutations in LRRK2 across the prodromal and early disease spectrum and in people with GBA1 mutations during the pre-symptomatic phase. There are multiple reasons why cholinergic function declines in PD: (1) “normal” age-related degeneration of the basal forebrain^32–34^ and cholinergic pathways^24^ and (2) additional degeneration due to PD-associated pathophysiological processes^35,36^. Although longitudinal imaging data are needed to confirm this, it appears that LRRK2 mutations are protective against/compensate for both kinds of degeneration, as evidenced by larger volumes (1) compared to similarly aged healthy non-mutation carriers (pointing toward less age-related degeneration) and (2) compared to non-mutation carriers with PD (indicating less PD-related degeneration). GBA1 mutations, on the other hand, might only have an effect on age-, but not disease-related degeneration. Whether this effect is protective or compensatory and the underlying physiological mechanisms remain to be investigated. A third possibility would be a pathological swelling of the basal forebrain due to a mutation-related developmental disorder that starts with the beginning of brain development and is later reversed by neurodegenerative processes in GBA1 but not LRRK2 mutation carriers.

A potential explanation for the differences between LRRK2 and GBA1 mutation carriers could be differences in pathology. Almost all GBA1 mutation carriers show Lewy body pathology^37^ and GBA1 mutations are also a significant risk factor for dementia with Lewy bodies where they have been associated with a purer alpha-synucleinopathy with less AD co-pathology^38,39^. In contrast, in PD-LRRK2, the prevalence of Lewy body pathology is more variable and specifically related to the occurrence of cognitive impairment while a primarily motor phenotype can occur in the absence of alpha-synuclein pathology^37,40^. Based on this, the results of the present study would support previous studies suggesting a particular link between basal forebrain atrophy and Lewy body pathology and observations that cholinergic deficits can be more severe in people with Lewy body disease compared to AD^41–43^. CSF biomarkers of AD co-pathology were not different between the groups (except for higher p-tau_181_ values in controls compared to asymptomatic GBA1 carriers), indicating that the observed group differences within the cholinergic system are largely independent of AD pathological processes.

Non-manifesting carriers of genetic mutations offer a unique population for investigation of potential disease-modifying interventions. This is particularly pertinent in the context of the cholinergic system in light of new approaches for treatment of basal forebrain degeneration^44–46^. This might have clinical relevance particularly in GBA1 mutation carriers who present with an intact cholinergic system at asymptomatic stages, but cholinergic degeneration comparable to or even worse than^22^ idiopathic PD patients at the symptomatic stage. Early identification of GBA1 mutation carriers who are at increased risk of cholinergic decline might therefore offer a window for intervention to ameliorate or prevent cholinergic degeneration and ultimately cognitive decline. However, it should be noted that GBA1 and LRRK2 mutations have variable and age-dependent lifelong penetrance^10,47,48^ and identifying those individuals that will develop symptoms is an important task for future research. So far, in the present sample only four of the asymptomatic mutation carriers have developed PD during the follow-up, precluding an analysis of the predictive power of basal forebrain volume for phenoconversion. However, considering the subgroup analysis in which we stratified asymptomatic individuals based on DATscan positivity can be helpful assuming that those individuals with positive DATscans are at an increased risk of having prodromal PD and converting to PD in the future^49^. We found that GBA1 mutation carriers with abnormal DATscans already show smaller basal forebrain volumes which was not the case in the LRRK2 mutation carriers, indicating a progressive degeneration of the basal forebrain in GBA1, but not LRRK2 mutation carriers, from asymptomatic to PD stages.

This study has some limitations. T1-weighted imaging data in PPMI were acquired at different field strengths, on different scanner models, and with different acquisition protocols. In this study, we aimed to include as much data as possible across different scanners, field strengths, and protocols. However, to test the robustness of the results we also performed a sensitivity analysis restricted to data from 3T Siemens scanners with results largely consistent with the analysis of the full sample. The only difference was that in this subset analysis the basal forebrain volume of the PD-GBA1 patients was higher (more similar to the PD-LRRK2 group) than in the full sample. This might be related to the fact that those PD-GBA1 participants with 3T Siemens data tended to be less cognitively impaired than the remaining PD-GBA1 patients (mean MoCA score of 27.2 (SD = 2.4) vs. 25.8 (SD = 2.6), BF_−0_ = 3.2).

For the DWI analysis, while acquisition protocols were more harmonised, the sample size was relatively small and the findings should therefore be tested in a larger sample with high-quality DWI data. Fitting a bi-tensor model to single-shell diffusion data requires some regularisations, and more advanced multi-shell acquisitions are still needed to test the reliability of these methods^50^. However, regardless of the biological interpretation of the estimated diffusion metrics, a recent study suggests that a bi-tensor model fit to single-shell data still exhibits improved signal-to-noise and contrast-to-noise properties over the standard single-tensor DTI model, especially when studying age-related changes in white matter structures^51^.

In conclusion, while LRRK2 and GBA1 mutations are both associated with a specific increase in basal forebrain volume at the asymptomatic stage, this increase persists at the symptomatic PD stage only in LRRK2 carriers and might be related to the slower rate of cognitive decline in these patients.

## Methods

### Participants

Data used in the preparation of this article were openly available from the Parkinson’s Progression Markers Initiative (PPMI) database (www.ppmi-info.org/access-data-specimens/download-data). For up-to-date information on the study, visit www.ppmi-info.org. We included participants from six groups: Idiopathic PD (defined herein as PD without mutations in GBA1, LRRK2, SNCA, Parkin or PINK1), LRRK2 mutation carriers with PD, GBA1 mutation carriers with PD, asymptomatic LRRK2 and GBA1 mutation carriers, and healthy controls. The idiopathic PD group comprises individuals aged 30 years or older with early untreated PD, i.e. a diagnosis of PD for 2 years or less, Hoehn and Yahr stage I or II, and not taking levodopa, dopamine agonists, MAO-B inhibitors, amantadine or another PD medication at baseline. PD participants with GBA1 or LRRK2 mutations were included if they had a PD diagnosis for 7 years or less, Hoehn and Yahr stage <4 and the respective mutation confirmed by genetic testing with no restriction on the use of PD medication. Asymptomatic GBA1 and LRRK2 mutation carriers were required to be 45 years or older. The healthy control group recruited participants aged 30 years or older without any significant neurological disorder and no first-degree relative with PD.

We included all PPMI participants from these six groups that were included in the PPMI analytic dataset from September 2022 with the following constraints:

1. Good quality T1-weighted MRI scan: participants whose scan had a quality rating of 3 (“lower quality”) were excluded. Additionally, all scans were visually checked and those with large artefacts were excluded.
2. Control participants were restricted to those who were 45 years or older as this was the inclusion criterion for asymptomatic GBA1 and LRRK2 mutation carriers.
3. Time between the baseline clinical visit and the MRI scan <6 months.
4. Participants who had mutations in both GBA1 and LRRK2 or additional mutations in SNCA, Parkin or PINK1 were excluded.

After applying these criteria, we included 149 asymptomatic GBA1 mutation carriers, 169 asymptomatic LRRK2 mutation carriers, 112 LRRK2 mutation carriers with PD, 60 GBA1 mutation carriers with PD, 492 idiopathic PD participants, and 180 healthy controls. The most common type of LRRK2 mutation was G2019S which was present in 133 of the asymptomatic carriers and 93 of the PD-LRRK2 patients, followed by the R1441G mutation (13 asymptomatic carriers and 14 PD patients) and R1441C mutation (1 PD patient). In the GBA1 groups, most participants had the N409S mutation (49 PD and 161 asymptomatic carriers) while other mutations were less common (PD-GBA1: 3 L483P, 1 IVS2 + 1G > A, 2 L29Afs*18, 1 R502C, 1 T408M; asymptomatic GBA1: 1 IVS2 + 1G > A, 3 L29Afs*18, 4 L483P). Due to the low number of participants who were carriers of the less common mutations, i.e. non-G2019S for LRRK2 or non-N409S for GBA1, we treated all LRRK2 and all GBA1 mutation carriers as one group for the main analysis. In additional analyses, we restricted the analysis to the G2019S and N409S mutation carriers to test if results were comparable to the whole cohort.

At each centre participating in PPMI, the study was approved by the local ethics committee and was conducted in accordance with the Good Clinical Practice guidelines. Written informed consent was obtained from each participant prior to inclusion in the study.

### MRI acquisition and processing

T1-weighted 3D volumetric MR images in PPMI were acquired on different 1.5 or 3T scanners with a MPRAGE or IR-FSPGR sequence and slice thickness of 2 mm or less. T1-weighted MR images were segmented into grey matter, white matter, and cerebrospinal fluid, and spatially normalised to MNI space using the CAT12 toolbox in SPM12 (http://www.fil.ion.ucl.ac.uk/spm/). Voxel values of spatially normalised grey matter maps were modulated by the Jacobian determinant of the deformation parameters in order to preserve the volume present in native space. The volume of the basal forebrain was estimated from the normalised grey matter images by summing up the modulated grey matter values within a consensus ROI combining information from existing cytoarchitectonic maps of basal forebrain cholinergic nuclei in MNI space, which have been derived from combined histology and MRI of post-mortem brains^52–55^. We estimated the volume of two basal forebrain sub-regions that were identified based on their differential cortical connectivity profile in resting state fMRI data^53^. In this functionally defined subdivision, the posterior basal forebrain mainly corresponds to the cytoarchitectonic sub-region of the nucleus basalis of Meynert (NBM) while the anterior basal forebrain covers the medial septum and diagonal band of Broca (see Fig. 1a).

For comparison, hippocampal volume was determined from the T1-weighted magnetic resonance images using an analogous automated volumetry approach based on a consensus MNI template of the hippocampus according to the European Alzheimer’s Disease Consortium and Alzheimer’s Disease Neuroimaging Initiative (EADC-ADNI) Harmonized Protocol^56^. All regional volumes were normalised with respect to total intracranial volume for each participant and volumes for left and right hemispheres were averaged.

To further test the specificity of findings for the basal forebrain, we additionally performed a whole-brain voxel-based morphometry (VBM) analysis in SPM using covariates for age, sex, education (+disease duration for PD groups), and total intracranial volume. The normalised, modulated grey matter images were smoothed with a Gaussian kernel of 8 mm full-width at half-maximum prior to the voxelwise analysis. Results from the voxelwise analysis were family-wise error (FWE)-corrected for multiple comparisons.

As a sensitivity analysis we restricted data to those from 3T Siemens scanners (99 controls, 68 asymptomatic GBA1, 76 asymptomatic LRRK2, 289 idiopathic PD, 28 PD-GBA1, and 63 PD-LRRK2) and repeated all analyses.

### DWI acquisition and processing

We included all DWI data from 3T Siemens scanners with 64 gradient directions with *b* = 1000 s/mm^2^ and one *b* = 0 s/mm^2^ image. Data were available for 53 healthy controls, 18 asymptomatic GBA1 carriers, 26 asymptomatic LRRK2 carriers, 16 PD-GBA1, 17 PD-LRRK2, and 132 idiopathic PD participants. DWI data were analysed using functions from the FMRIB software library (FSL) version 6.0.3. DWI data were brain extracted using FSL’s bet function prior to applying FSL’s eddy tool to correct for eddy currents and head motion^57^ and N4 bias field correction from Advanced Normalisation Tools (ANTs)^58^. FSL’s BedpostX was applied to the preprocessed DWI data to calculate the diffusion parameters using a standard ball-and-sticks model with three fibres modelled per voxel. A bi-tensor model was fit to the eddy- and bias-corrected diffusion data to estimate free water fraction (FWf) and free water-corrected mean diffusivity (cMD) and axial diffusivity (cAxD) within each voxel (https://github.com/mvgolub/FW-DTI-Beltrami)^59,60^.

### Tractography

We performed tractography of the main cholinergic pathways as described previously using FSL’s ProbtrackX by generating 5000 random samples from the respective seed regions of interest (NBM/PPN)^23–25^. From the NBM we examined a medial pathway travelling through the cingulum toward cingulate, retrosplenial and subcallosal cortex, and a lateral pathway travelling through the external capsule and uncinate fasciculus to innervate the insula, frontal, parietal and temporal cortex. From the PPN we studied pathways to the thalamus. For the PPN seed region, we used a stereotactic map that had been derived from combined histology and post-mortem MRI of the brain of a 66-year-old woman who showed no signs of Parkinsonism or cognitive decline^61^. Seed region maps were transformed to native space using ANTs. A detailed description of the tractography methods can be found in Schumacher et al.^23^ and Schumacher et al.^25^. Briefly, the respective tracts were estimated in every participant, transformed to a common group template space, and thresholded (retaining voxels that were part of the tract in at least 50% of participants for the NBM tracts and at least 70% of participants for the PPN-thalamus tract, see Fig. 2a). After transforming the group tracts back into subject space, mean FWf, cMD and cAD were extracted from all voxels belonging to the respective tract. Furthermore, we extracted diffusivity metrics from the PPN itself. Since the PPN has white matter pathways from the brainstem running through it, this analysis was restricted to voxels with fractional anisotropy <0.77 based on values reported in Alho et al.^61^ (mean + 1 standard deviation). To control for general white matter changes, a white matter control mask was created by subtracting the respective tract from a whole-brain white matter mask that was obtained by running FSL FAST on each subject’s T1-weighted image. For the analysis of diffusivity metrics in the PPN, a grey matter control mask was created by subtracting the PPN ROI from a whole-brain grey matter mask.

Voxelwise group differences in diffusion metrics along the estimated tracts were assessed using permutation-based non-parametric testing with FSL’s randomise function, correcting for multiple comparisons using threshold-free cluster enhancement (TFCE) and including covariates for age, sex, years of education (and disease duration for the PD groups).

### Neuropsychological assessment

Participants underwent a detailed neuropsychological assessment covering different cognitive domains: Montreal Cognitive Assessment (MoCA, global cognition), Benton Judgement of Line Orientation (visuospatial function), Letter Number Sequencing (executive function/working memory), Symbol Digit Modalities Test (processing speed/attention), animal fluency test (executive function/language), and Hopkins Verbal Learning Test-Revised (HVLT, episodic memory). These assessments were conducted at baseline and during annual follow-up visits. We included data up to the 5-year follow-up visit in the longitudinal analyses.

### CSF biomarkers of Alzheimer’s pathology

Cerebrospinal fluid (CSF) was collected at each study site by standardised lumbar puncture procedures and shipment and storage was performed as described in the PPMI biologics manual (http://ppmi-info.org). The frozen aliquots of CSF were transferred from the PPMI Biorepository Core laboratories to the University of Pennsylvania for analyses. Data were analysed using the Elecsys Amyloid-beta(1–42), t-tau and p-tau181 electrochemiluminescence immunoassays on a fully automated cobas e601 analyser (Roche Diagnostics).

### DATscan imaging

DATscan imaging with [123I]-FP-CIT SPECT was performed at each imaging centre and sent to the PPMI imaging core lab at the Institute for Neurodegenerative Disorders (IND) for visual interpretation by two expert readers. Scans were read as either showing evidence of dopamine transporter deficit (i.e. positive DATscan) or not showing evidence of dopamine transporter deficit (i.e. negative DATscan).

### Statistical analysis

All statistical analyses were performed in a Bayesian framework. Bayesian statistics is an approach to parameter estimation based on Bayes’ theorem where prior knowledge about parameters of a statistical model is updated with the information obtained from observed data. A Bayesian analysis therefore consists of deriving a posterior distribution of the parameters of interest from the combination of a prior distribution and the model likelihood estimated from the data^62^. This framework is increasingly being adopted across many scientific fields including medical research thanks to its advantages over the classical frequentist approach which include the ability to directly compare several hypotheses and the possibility of a quantitative interpretation of evidence beyond the classical dichotomisation into significant and non-significant^63^.

We used Jeffreys’s Amazing Statistics Program (JASP, version 0.17.1) for group comparisons. Numerical accuracy was established with 10,000 iterations using a Markov Chain Monte Carlo algorithm. In addition to the posterior distribution of parameter estimates, we report the Bayes Factor (BF_10_)^64^ to quantify evidence in favour of the alternative over the null hypothesis^65^. The Bayes Factor is interpreted as the relative likelihood of the data under the models of interest, i.e. BF_10_ quantifies the likelihood of the data given H1 compared to the likelihood of the data given H0. According to Bayesian analysis reporting guidelines in JASP^66^, a BF_10_ between 3 and 10 indicates a moderate, a BF_10_ between 10 and 30 indicates a strong, a BF_10_ between 30 and 100 indicates a very strong, and a BF_10_ > 100 indicates an extreme level of evidence in favour of the alternative model over the null model. Equivalently, if BF_10_ is below 1/3, 1/10, 1/30, 1/100, it indicates a moderate, strong, very strong, or extreme level of evidence, respectively, in favour of the null over the alternative hypothesis. Thus, a key strength of Bayesian hypothesis testing as opposed to the frequentist approach is that it provides the possibility to directly quantify support in favour of the null hypothesis, not only against it^65^.

We were particularly interested in two group comparisons: (1) asymptomatic LRRK2 mutation carriers vs. GBA1 mutation carriers vs. healthy controls, (2) idiopathic PD vs. PD-LRRK2 vs. PD-GBA1. As secondary analyses, we also compared the PD groups to healthy controls and the asymptomatic mutation carriers to the respective mutation carrier group with PD.

Anterior and posterior basal forebrain volumes as well as mean diffusion metrics within the PPN and along the NBM and PPN tracts were compared between the groups using Bayesian ANCOVAs including covariates for age, sex, and years of education. For the PD group comparison, disease duration was included as an additional covariate. We used the JASP default JZS prior for coefficients in all Bayesian ANCOVA models.

To further stratify the asymptomatic mutation carriers with respect to their probability of having prodromal PD, we subdivided each group into those with positive and negative DATscans. We then compared posterior and anterior basal forebrain volume between the subgroups using Bayesian ANCOVAs, including covariates for age, sex, and years of education.

To assess the influence of PD-related medication on imaging measures in the PD-GBA1 and PD-LRRK2 groups, we compared volumetric and diffusion measures between patients who were taking dopaminergic medication and those who were not.

Longitudinal changes in cognition over 5 years were assessed with Bayesian mixed models in the different groups using the cognitive scores as dependent variables, and time, age, sex, and years of education (and disease duration for PD groups) as fixed effects. The model included all main effects and random effects for intercepts and time. The effect of baseline basal forebrain volume on changes in cognition was assessed with Bayesian mixed models with volume as an additional fixed effect and including the interaction between time and volume in the model. All Bayesian mixed models were run with the brms package (version 2.20.1)^67^ in R (https://www.r-project.org/). As the calculation of Bayes factors for these models is not as straightforward and still debated^68^, we show the posterior distribution of the parameter estimates instead and report the median and 95% credible intervals. While the Bayesian framework does not include a notion of “statistical significance”, we would interpret a posterior distribution whose 95% credible interval does not include zero as evidence for an effect. The central tendency of the posterior distribution for a parameter can be interpreted like the parameter estimate for the respective effect in a standard frequentist mixed model. The Bayesian 95% credible interval represents the bounds within which the true value of the parameter is expected to lie with 95% probability. In contrast, for the frequentist 95% confidence interval, “[o]ver infinite repeated sampling, […], the [95%]-level confidence interval will include the true value in [95%] of the samples for which it is calculated”^69^; i.e. the confidence interval relates to long-term realisations of the parameter value in future hypothetical experiments. The direct interpretability of the Bayesian credible interval compared with the frequentist confidence interval is another advantage of Bayesian analysis.

### Supplementary information

Supplementary Material

## Data Availability

Data used in the preparation of this article were obtained on March 6, 2023 from the Parkinson’s Progression Markers Initiative (PPMI) database (www.ppmi-info.org/access-dataspecimens/download-data), RRID:SCR_006431. For up-to-date information on the study, visit www.ppmi-info.org.
