# Supplementary material for: Associations of cholinergic system integrity with cognitive decline in GBA1 and LRRK2 mutation carriers
Source: NPJ Parkinsons Dis. 2024 Jun 29;10:127. doi: 10.1038/s41531-024-00743-w (PMC11217433; doi:10.1038/s41531-024-00743-w)
Supplement: Supplementary file 1 — Supplementary Material [file 41531_2024_743_MOESM1_ESM.pdf]

Supplementary Material

Associations of cholinergic system integrity with cognitive decline in GBA1 and LRRK2 mutation carriers

Julia Schumacher, Nicola Ray, Stefan Teipel, Alexander Storch

Correspondence to: julia.schumacher@dzne.de

Contents

1. Demographics of DWI subsample
2. Volumetric group comparisons
    2.1. Posterior distributions
    2.2. Effects of MRI field strength
    2.3. Analyses of genetic subgroups
3. Effects of Parkinson's medication
4. DWI group comparisons
    4.1. Posterior distributions
    4.2. Sequential analysis
    4.3. Voxelwise analysis
5. Longitudinal analysis
    5.1. Longitudinal cognitive scores
    5.2. Associations with anterior basal forebrain volume
6. Frequentist analysis
    6.1. Group comparisons
    6.2. Longitudinal associations with cognition

1. Demographics of DWI subsample

Supplementary Table 1: Demographic and clinical information for the DWI subsample. Mean (standard deviation).

	Control (N=53)	Asymptomatic GBA1 (N=18)	Asymptomatic LRRK2 (N=26)	Group comparison
Age at baseline	62.5 (8.1)	62.0 (8.4)	59.4 (5.9)	BF_{10}=0.35[a]
Male:female	32:21	8:10	11:15	BF_{10}=0.33[b]
Years of education	15.8 (3.0)	18.4 (2.8)	18.1 (3.1)	BF_{10}=41.9[a], HC<GBA1, LRRK2
MoCA	28.2 (1.1)	27.2 (1.7)	27.9 (1.3)	BF_{10}=2.5[a]
UPDRS III (OFF)	0.7 (1.4)	3.4 (6.3)	1.9 (3.5)	BF_{10}=3.8[a], GBA1>HC, LRRK2
	**Idiopathic PD (N=132)**	**PD GBA1 (N=16)**	**PD LRRK2 (N=17)**	**Group comparison**
Age at baseline	61.4 (9.4)	66.5 (8.9)	61.5 (11.5)	BF_{10}=0.60[a]
Male:female	86:46	10:6	10:7	BF_{10}=0.03[b]
Years of education	15.2 (3.0)	16.4 (2.3)	16.9 (3.3)	BF_{10}=1.49[a]
MoCA	27.6 (2.1)	26.4 (2.3)	27.5 (2.0)	BF_{10}=0.81[a]
UPDRS III (OFF)	20.3 (8.8)	26.2 (12.0)	24.6 (11.5)	BF_{10}=2.57[a]
Disease duration (in years)	0.54 (0.54)	1.99 (2.02)	2.24 (1.87)	$BF_{10}=2.6*10^{10}$, iPD<GBA1, LRRK2[a]

[a] Bayesian ANOVA all groups (with post-hoc tests), [b] Bayesian contingency tables test
iPD, idiopathic Parkinson's disease; MoCA, Montreal Cognitive Assessment; UPDRS, Unified Parkinson's disease rating scale

2. Volumetric group comparisons

2.1. Posterior distributions

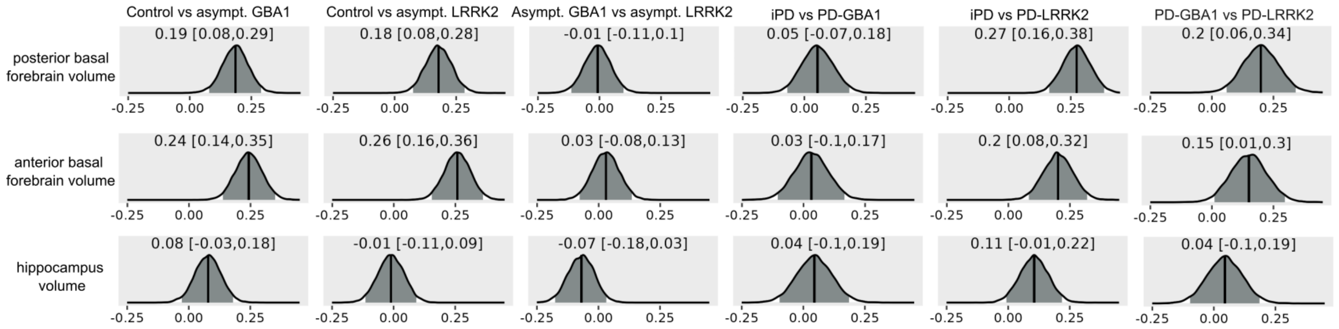

Supplementary Figure 1: Posterior distributions of parameter estimates for the effect of group in comparisons of basal forebrain and hippocampus volume (normalized with respect to total intracranial volume) by Bayesian ANCOVAs, including covariates for age, sex, years of education, and disease duration (only for PD groups). The median is marked with a solid line and the 95% credible intervals in grey, and stated in each plot.

2.3. Effects of MRI field strength

Supplementary Table 2: Group comparisons of basal forebrain and hippocampus volume by Bayesian ANCOVAs, including covariates for age, sex, years of education, and disease duration (only for PD groups), restricted to data from 3T Siemens scanners (including 99 controls, 68 asymptomatic GBA1, 76 asymptomatic LRRK2, 289 idiopathic PD, 28 PD-GBA1, and 63 PD-LRRK2).

	Posterior basal forebrain volume	Anterior basal forebrain volume	Hippocampus volume
HC vs asympt. GBA1	**BF_{10}=301.8**	**BF_{10}=1.1x10^{5}**	BF_{10}=0.57
HC vs asympt. LRRK	**BF_{10}=279.5**	**BF_{10}=10630**	BF_{10}=0.24
Asympt. GBA1 vs asympt. LRRK2	BF_{10}=0.26	BF_{10}=0.31	BF_{10}=0.41
iPD vs PD-GBA1	BF_{10}=2.5	**BF_{10}=3.4**	BF_{10}=1.2
iPD vs PD-LRRK2	**BF_{10}=11850**	**BF_{10}=44.2**	BF_{10}=0.85
PD-GBA1 vs PD-LRRK2	BF_{10}=0.39	BF_{10}=0.46	BF_{10}=0.31
HC vs iPD	**BF_{10}=9.8**	BF_{10}=0.13	BF_{10}=0.75
HC vs PD-GBA1	BF_{10}=0.26	BF_{10}=1.8	BF_{10}=0.26
HC vs PD-LRRK2	BF_{10}=1.16	**BF_{10}=56.6**	BF_{10}=0.19
Asympt. GBA1 vs PD-GBA1	BF_{10}=1.69	BF_{10}=0.84	BF_{10}=0.32
Asympt. LRRK2 vs PD-LRRK2	BF_{10}=0.91	BF_{10}=0.32	BF_{10}=0.19

BF, basal forebrain; BF_{10}, Bayes factor quantifying evidence against the null hypothesis; HC, healthy controls; iPD, idiopathic Parkinson's disease

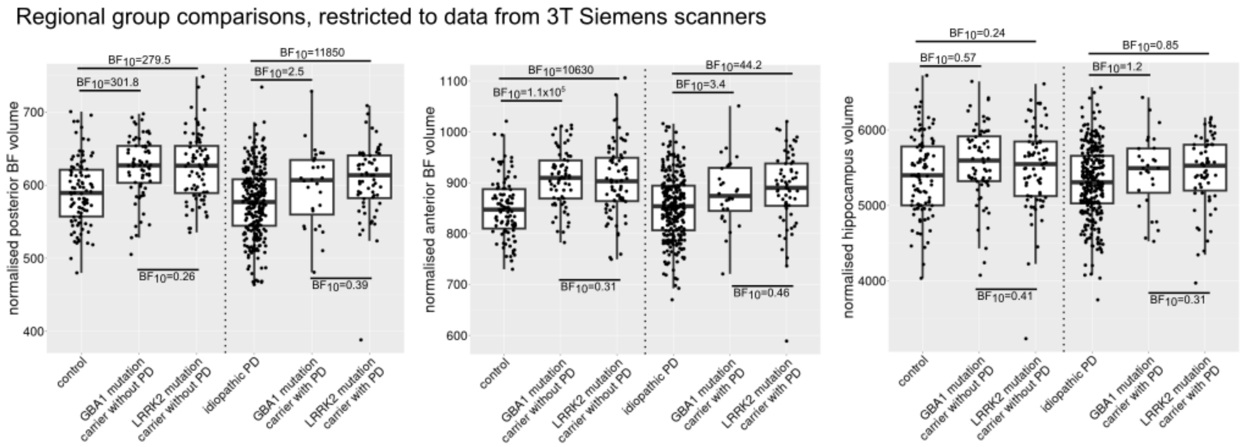

Supplementary Figure 2: Group comparison of subregional basal forebrain (BF) and hippocampus volume (normalized with respect to total intracranial volume), restricted to data from 3T Siemens scanners. Bayes factors quantifying evidence against the null hypothesis (BF_{10}) from Bayesian ANCOVAs including covariates for age, sex, years of education (and disease duration for the PD groups).

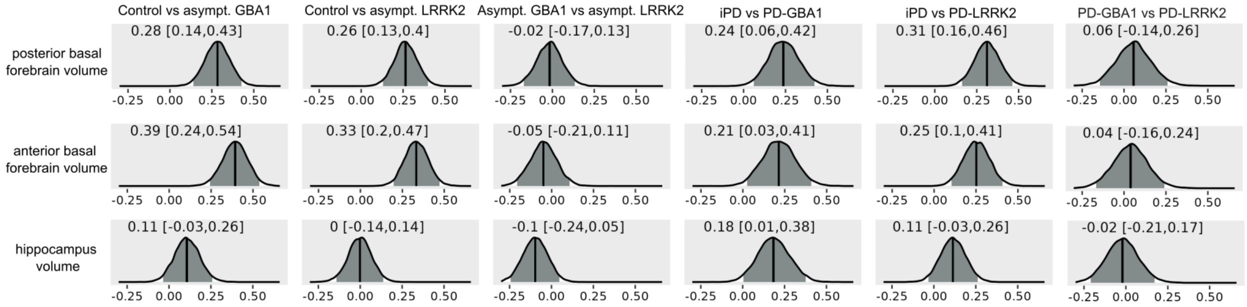

Supplementary Figure 3: Posterior distributions of parameter estimates for the effect of group in comparisons of basal forebrain and hippocampus volume (normalized with respect to total intracranial volume) by Bayesian ANCOVAs, including covariates for age, sex, years of education, and disease duration (only for PD groups), restricted to data from 3T Siemens scanners. The median is marked with a solid line and the 95% credible intervals in grey, and stated in each plot.

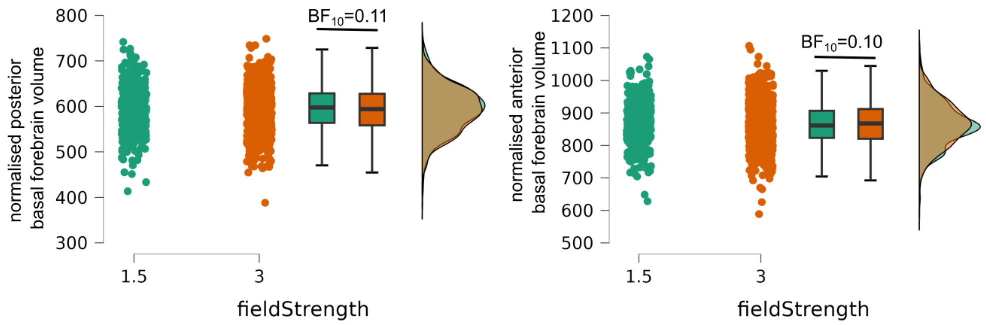

Supplementary Figure 4: Comparison of basal forebrain volumes from 1.5 and 3T scanners.
BF_{10}, Bayes factor quantifying evidence against the null hypothesis

2.2. Analyses of genetic subgroups

Supplementary Table 3: Analysis restricted to GBA1 mutation carriers with the N409S mutation and LRRK2 mutation carriers with the G2019S. Group comparisons of basal forebrain and hippocampus volume (normalized with respect to total intracranial volume) by Bayesian ANCOVAs, including covariates for age, sex, years of education, and disease duration (only for PD groups).

	Posterior basal forebrain volume	Anterior basal forebrain volume	Hippocampus volume
HC vs asympt. GBA1	**BF_{10}=166.0**	**BF_{10}=8169.7**	BF_{10}=0.55
HC vs asympt. LRRK	**BF_{10}=23.2**	**BF_{10}=1949.8**	BF_{10}=0.13
Asympt. GBA1 vs asympt. LRRK2	BF_{10}=0.14	BF_{10}=0.13	BF_{10}=0.62
iPD vs PD-GBA1	BF_{10}=0.26	BF_{10}=0.24	BF_{10}=0.24
iPD vs PD-LRRK2	**BF_{10}=275.7**	BF_{10}=2.5	BF_{10}=0.53
PD-GBA1 vs PD-LRRK2	**BF_{10}=4.6**	BF_{10}=0.50	BF_{10}=0.31
HC vs PD-GBA1	BF_{10}=0.37	BF_{10}=0.26	BF_{10}=0.21
HC vs PD-LRRK2	BF_{10}=0.58	**BF_{10}=15.3**	BF_{10}=0.15
Asympt. GBA1 vs PD-GBA1	**BF_{10}=619.9**	BF_{10}=2.8	BF_{10}=1.1
Asympt. LRRK2 vs PD-LRRK2	BF_{10}=0.46	BF_{10}=0.29	BF_{10}=0.17

BF, basal forebrain; BF_{10}, Bayes factor quantifying evidence against the null hypothesis; HC, healthy controls; iPD, idiopathic Parkinson's disease

3. Effects of Parkinson's medication

Supplementary Table 4: Comparison of volumetric and diffusivity measures between PD patients who are taking and those who are not taking PD medication by Bayesian ANCOVAs including covariates for age, sex, years of education (and diffusivity metric from the respective control mask for the diffusivity metrics).

	PD-GBA1 taking PD meds (N=46) vs PD-GBA1 not taking PD meds (N=14)	PD-LRRK2 taking PD meds (N=100) vs PD-LRRK2 not taking PD meds (N=12)
Posterior basal forebrain volume	BF_{10}=0.39	BF_{10}=0.30
Anterior basal forebrain volume	BF_{10}=0.50	BF_{10}=0.37
Hippocampus volume	BF_{10}=0.39	BF_{10}=0.34
FW NBM lateral tract	BF_{10}=0.66	BF_{10}=0.61
FWc MD NBM lateral tract	BF_{10}=0.90	BF_{10}=0.70
FWc AD NBM lateral tract	BF_{10}=0.47	BF_{10}=1.86
FW NBM medial tract	BF_{10}=0.49	BF_{10}=0.69
FWc MD NBM medial tract	BF_{10}=0.50	BF_{10}=0.64
FWc AD NBM medial tract	BF_{10}=0.51	BF_{10}=0.63
FW PPN	BF_{10}=0.49	BF_{10}=0.59
FWc MD PPN	BF_{10}=0.48	BF_{10}=0.60
FWc AD PPN	BF_{10}=0.56	BF_{10}=0.59
FW PPN-thalamus tract	BF_{10}=0.56	BF_{10}=1.19
FWc MD PPN-thalamus tract	BF_{10}=0.53	BF_{10}=0.65
FWc AD PPN-thalamus tract	BF_{10}=0.64	BF_{10}=0.60

AD, axial diffusivity; BF_{10}, Bayes factor quantifying evidence against the null hypothesis; FW, free water fraction; FWc, free water-corrected; MD, mean diffusivity; NBM, nucleus basalis of Meynert; PD, Parkinson's disease; PPN, pedunculopontine nucleus

4. DWI group comparisons

4.1. Posterior distributions

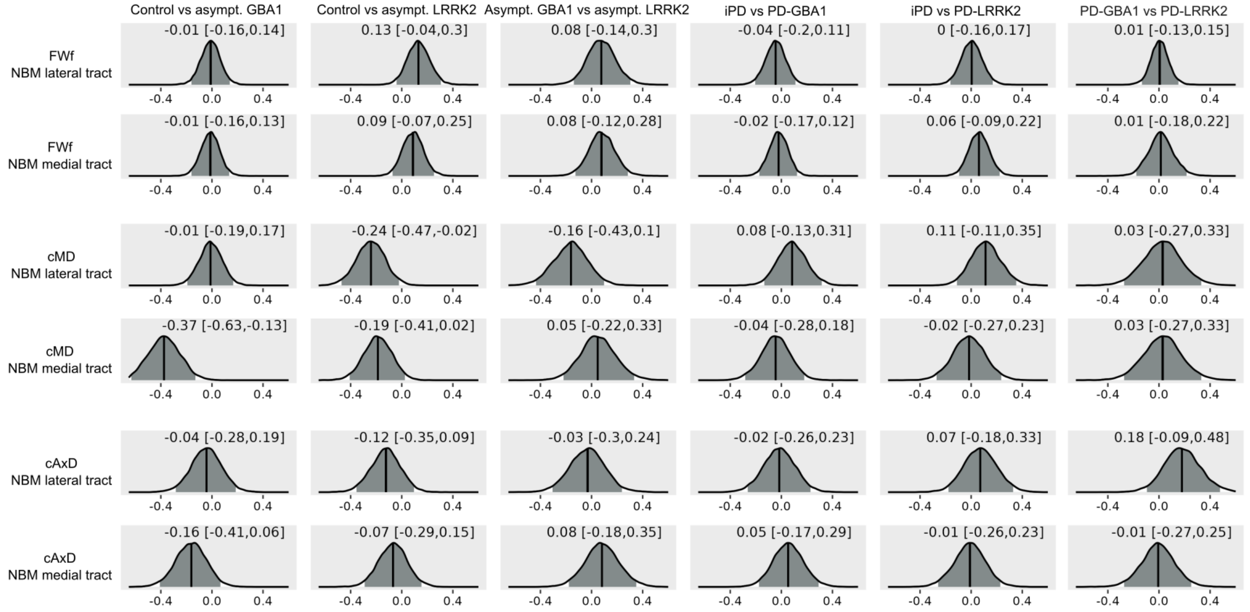

Supplementary Figure 5: Group comparison of free water DTI metrics in NBM pathways. Posterior distributions of parameter estimates for the effect of group in comparisons of free water DTI metrics by Bayesian ANCOVAs, including covariates for age, sex, years of education, and disease duration (only for PD groups). The median is marked with a solid line and the 95% credible intervals in grey, and stated in each plot.
cAxD, free water-corrected axial diffusivity; cMD, free water-corrected mean diffusivity; FWf, free water fraction; NBM, nucleus basalis of Meynert; PD, Parkinson’s disease.

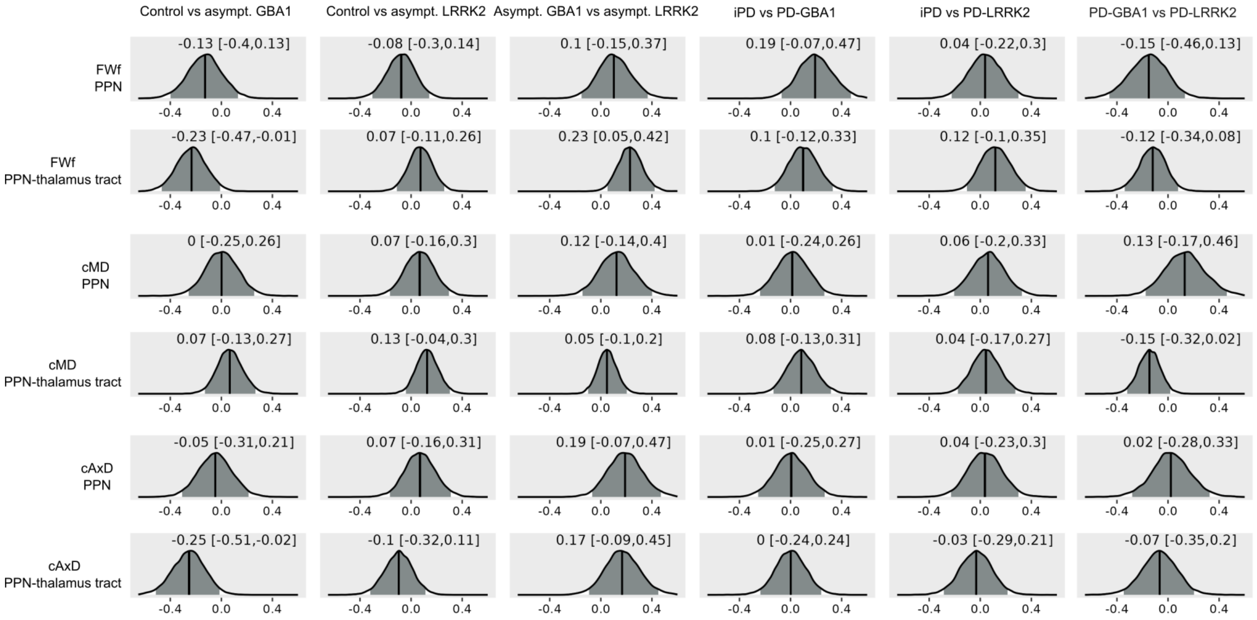

Supplementary Figure 6: Group comparison of free water DTI metrics in PPN and PPN-thalamus pathway. Posterior distributions of parameter estimates for the effect of group in comparisons of free water DTI metrics by Bayesian ANCOVAs, including covariates for age, sex, years of education, and disease duration (only for PD groups). The median is marked with a solid line and the 95% credible intervals in grey, and stated in each plot. cAxD, free water-corrected axial diffusivity; cMD, free water-corrected mean diffusivity; FWf, free water fraction; PD, Parkinson’s disease; PPN, pedunculopontine nucleus.

4.2. Sequential analysis

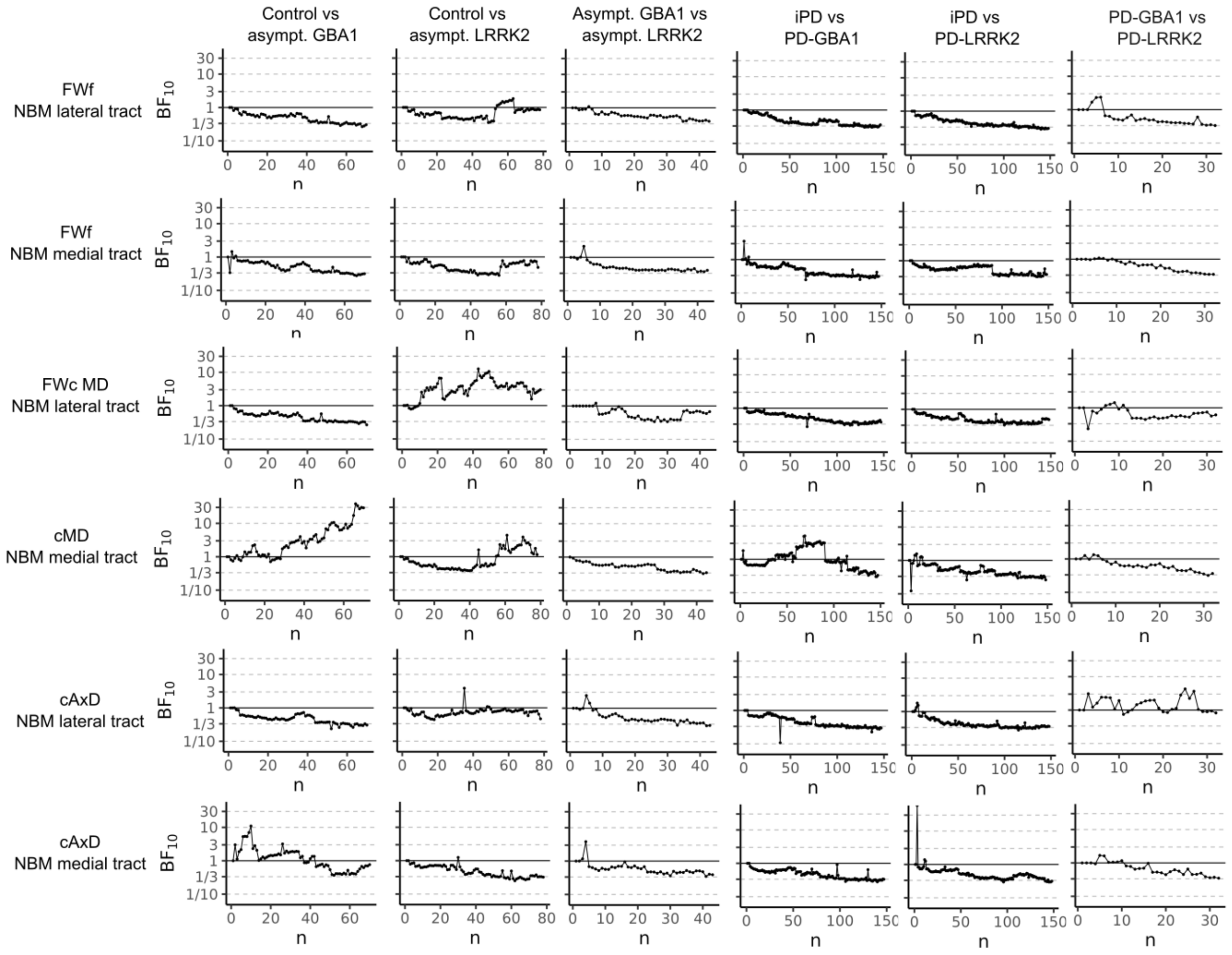

Supplementary Figure 7: Group comparison of free water DTI metrics in NBM pathways by Bayesian ANCOVAs, including covariates for age, sex, years of education, and disease duration (only for PD groups). Sequential analysis evaluating the Bayes Factor after sequentially adding one observation at a time. The horizontal line marks a BF_{10} of 1, indicating equal support for the null and the alternative hypothesis.

cAxD, free water-corrected axial diffusivity; cMD, free water-corrected mean diffusivity; FWf, free water fraction; NBM, nucleus basalis of Meynert; PD, Parkinson's disease.

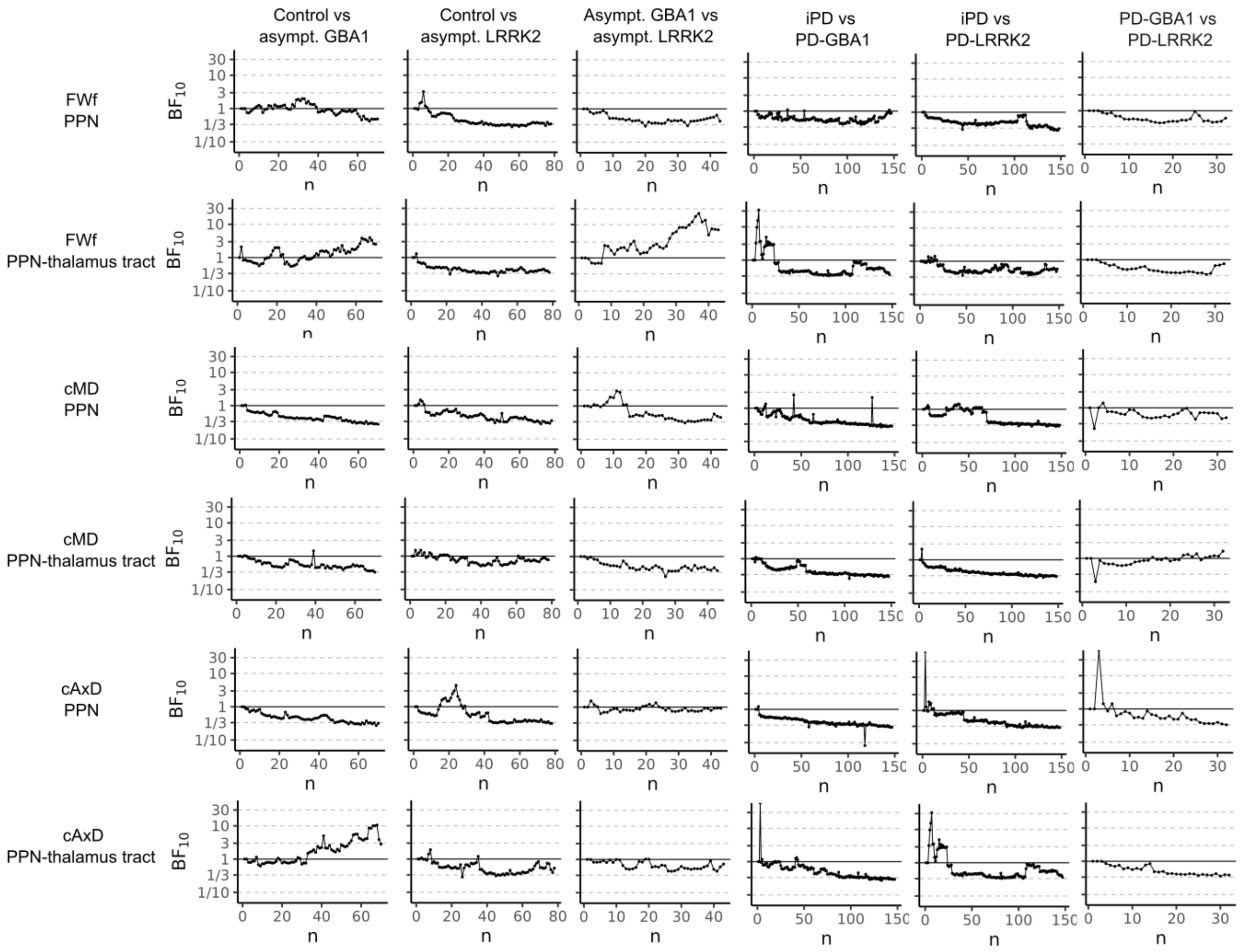

Supplementary Figure 8: Group comparison of free water DTI metrics in PPN and PPN-thalamus pathway by Bayesian ANCOVAs, including covariates for age, sex, years of education, and disease duration (only for PD groups). Sequential analysis evaluating the Bayes Factor after sequentially adding one observation at a time. The horizontal line marks a BF_{10} of 1, indicating equal support for the null and the alternative hypothesis.
cAxD, free water-corrected axial diffusivity; cMD, free water-corrected mean diffusivity; FWf, free water fraction; PD, Parkinson's disease; PPN, pedunculopontine nucleus.

4.3. Voxelwise analysis

Supplementary Table 5: Results from voxelwise group comparisons of free water DTI metrics along NBM and PPN pathways using FSL-randomise. Shown are all clusters with TFCE-corrected $p<0.05$ with their size (number of voxels), p-value, and location (MNI coordinates X, Y, Z). All group comparisons that are not listed did not show any significant clusters.
cAxD, free water-corrected axial diffusivity; cMD, free water-corrected mean diffusivity; FWf, free water fraction; NBM, nucleus basalis of Meynert; PD, Parkinson's disease; PPN, pedunculopontine nucleus; TFCE, threshold-free cluster enhancement

**size**	**p_{TFCE}**	**X**	**Y**	**Z**	**size**	**p_{TFCE}**	**X**	**Y**	**Z**	**size**	**p_{TFCE}**	**X**	**Y**	**Z**
**FWf**					**cMD**					**cAxD**				
**NBM lateral pathway**														
Control > asymptomatic GBA1:														
8	0.024	40	56	30	11	0.016	60	66	30					
5	0.024	42	57	31	6	0.031	63	68	30					
4	0.024	45	45	29	3	0.017	62	70	30					
1	0.044	43	45	30	1	0.018	57	66	30					
					1	0.046	60	73	34					
Control > asymptomatic LRRK2:														
					2	0.043	67	33	35					
					1	0.045	40	40	32					
Idiopathic PD > PD LRRK2:														
9	0.014	62	44	29										
3	0.02	47	45	29										
PD LRRK2 > idiopathic PD:														
22	0.024	64	46	33										
2	0.035	61	51	37										
1	0.05	75	63	24										
1	0.046	69	46	33										
1	0.038	62	48	35										
**NBM medial pathway**														
Control > asymptomatic GBA1:														
3	0.022	42	54	29	4	0.016	60	66	30	5	0.001	59	66	46
1	0.027	41	57	30						2	0.019	59	68	45
Control > asymptomatic LRRK2:														
					3	0.018	53	65	31					
Idiopathic PD > PD LRRK2:														
3	0.021	59	62	29										
**PPN-thalamus pathway**														
Control > asymptomatic GBA1:														
1	0.007	50	53	30										
PD LRRK2 > idiopathic PD:														
1	0.04	54	50	24										
1	0.011	52	52	28										
1	0.001	60	53	29										
PD GBA1 > PD LRRK2:														
										1	0.045	61	51	28

5. Longitudinal analyses

5.1. Longitudinal cognitive scores

Supplementary Table 6: Mean (standard deviation) of cognitive scores per group and time point.

	Control	Asympt. GBA1	Asympt. LRRK2	Idiopathic PD	PD-GBA1	PD-LRRK2
MoCA						
BL	28.0 (1.4)	26.8 (2.2)	27.1 (2.2)	27.0 (2.4)	26.5 (2.6)	26.0 (3.1)
Year 1	27.4 (2.1)	26.8 (2.3)	27.5 (2.2)	26.6 (2.7)	25.6 (3.7)	25.8 (3.3)
Year 2	27.2 (2.4)	26.7 (2.6)	27.3 (2.3)	26.3 (3.1)	25.7 (3.4)	26.4 (2.8)
Year 3	27.6 (2.1)	27.1 (2.5)	27.8 (2.2)	26.4 (2.9)	26.3 (3.5)	26.2 (3.3)
Year 4	27.7 (2.2)	27.5 (1.9)	28.0 (1.9)	26.5 (3.4)	25.8 (4.3)	26.3 (3.2)
Year 5	27.6 (2.2)	27.2 (2.0)	27.5 (1.8)	26.8 (3.2)	25.6 (4.0)	26.5 (3.6)
Judgment of line orientation						
BL	26.5 (3.7)	25.9 (3.9)	25.8 (4.1)	25.7 (4.4)	24.7 (4.5)	23.8 (5.2)
Year 1	25.5 (4.9)	25.9 (4.2)	25.6 (4.1)	24.9 (4.7)	22.9 (4.9)	23.5 (5.1)
Year 2	26.4 (3.8)	26.2 (3.5)	26.3 (3.7)	25.6 (4.5)	24.1 (4.7)	24.3 (5.2)
Year 3	25.4 (4.4)	26.0 (4.3)	25.7 (3.5)	25.2 (4.6)	24.2 (5.3)	23.9 (4.6)
Year 4	26.0 (4.9)	28.2 (2.5)	26.3 (3.5)	25.9 (4.3)	23.8 (6.6)	24.4 (4.6)
Year 5	25.7 (4.3)	-	25.3 (4.8)	24.9 (4.6)	23.5 (6.4)	23.9 (5.0)
Letter number sequencing						
BL	10.8 (2.5)	11.6 (2.9)	11.2 (2.7)	10.6 (2.7)	10.9 (2.6)	10.0 (2.7)
Year 1	10.8 (2.5)	11.6 (2.7)	11.6 (2.8)	10.4 (2.6)	10.4 (3.3)	9.7 (2.8)
Year 2	10.8 (2.3)	11.9 (2.9)	11.4 (2.8)	10.2 (2.8)	10.2 (3.6)	10.2 (2.5)
Year 3	10.9 (2.7)	11.4 (2.6)	12.0 (2.9)	10.2 (2.9)	10.5 (4.2)	9.8 (2.3)
Year 4	11.0 (2.5)	11.6 (2.3)	12.6 (3.1)	10.1 (3.2)	9.7 (4.2)	9.8 (2.6)
Year 5	11.0 (2.6)	-	13.1 (2.8)	10.1 (2.9)	10.4 (5.0)	9.7 (2.9)
Symbol digit modalities test						
BL	46.9 (10.0)	47.6 (8.4)	46.9 (9.5)	41.8 (9.8)	41.3 (10.5)	40.3 (11.5)
Year 1	46.9 (9.1)	49.8 (8.9)	48.3 (10.4)	41.1 (10.2)	38.9 (12.7)	41.0 (12.2)
Year 2	45.5 (9.2)	49.2 (8.7)	46.5 (11.1)	40.3 (11.0)	39.1 (12.5)	39.9 (11.0)
Year 3	47.2 (9.4)	50.1 (9.6)	49.2 (10.4)	40.3 (11.5)	40.3 (13.5)	41.1 (13.5)
Year 4	45.6 (9.2)	51.4 (10.6)	49.9 (10.4)	39.4 (11.8)	38.0 (16.8)	38.8 (12.3)
Year 5	47.5 (10.4)	50.7 (9.3)	48.0 (9.3)	40.1 (12.2)	38.4 (16.4)	39.1 (12.7)
Semantic fluency – animals						
BL	22.4 (5.4)	23.7 (5.7)	23.7 (5.8)	21.1 (5.4)	22.8 (5.6)	20.8 (6.2)
Year 1	22.5 (5.3)	23.8 (5.7)	24.0 (6.4)	20.9 (5.4)	22.3 (6.1)	21.6 (6.2)
Year 2	22.4 (5.2)	23.8 (5.9)	23.9 (5.9)	20.9 (5.6)	21.5 (6.6)	20.5 (6.1)
Year 3	22.5 (5.4)	23.2 (5.8)	23.8 (6.4)	20.8 (5.2)	20.9 (6.4)	20.7 (5.5)
Year 4	21.8 (4.9)	23.2 (4.9)	23.7 (5.6)	21.0 (5.5)	20.4 (5.7)	20.0 (5.9)
Year 5	22.6 (5.5)	24.1 (5.8)	24.2 (6.2)	21.4 (5.9)	20.1 (6.3)	20.3 (6.5)
HVLT – delayed recall						
BL	9.1 (2.3)	9.6 (2.1)	9.7 (2.3)	8.3 (2.6)	8.2 (3.0)	8.2 (2.8)
Year 1	9.1 (2.4)	9.8 (2.3)	9.9 (2.0)	8.0 (2.8)	8.1 (2.7)	8.6 (2.8)
Year 2	9.1 (2.5)	9.8 (2.3)	9.9 (2.0)	8.1 (2.9)	7.7 (3.5)	8.6 (3.1)
Year 3	9.2 (2.4)	9.0 (3.0)	9.2 (2.4)	8.4 (3.0)	8.7 (2.6)	8.0 (3.1)
Year 4	9.1 (2.6)	8.9 (2.9)	9.5 (2.5)	8.2 (3.1)	6.6 (3.2)	8.5 (2.9)
Year 5	9.7 (2.7)	8.9 (3.3)	9.3 (3.2)	8.6 (3.2)	5.9 (4.0)	8.6 (3.2)

HVLT, Hopkins Verbal Learning Test; MoCA, Montreal Cognitive Assessment; PD, Parkinson’s disease

5.2. Associations with anterior basal forebrain volume

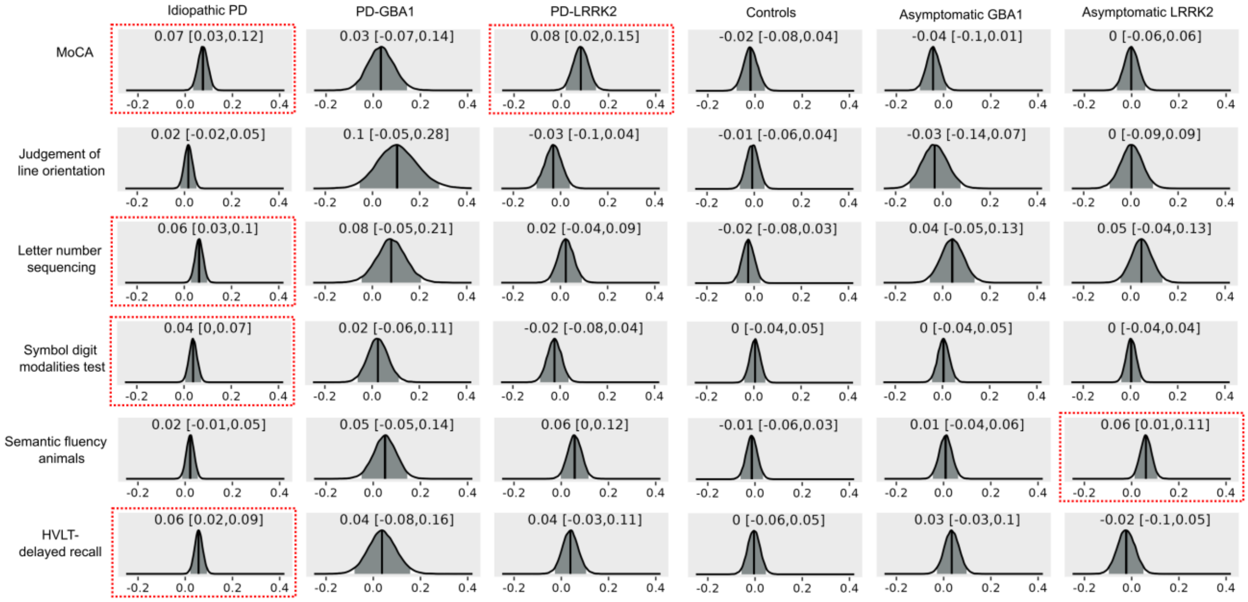

Supplementary Figure 9: Association between anterior basal forebrain volume and changes in cognition. Posterior distributions of parameter estimates for the interaction of time and anterior basal forebrain volume in Bayesian mixed models including cognitive scores as dependent variable, fixed effects for posterior basal forebrain volume, time, age, sex, years of education (and disease duration for PD groups), the interaction between time and anterior basal forebrain volume, and random effects for intercepts and time. The median is marked with a solid line and the 95% credible intervals in grey, and stated in each plot. Parameter estimates for which the 95% credible interval does not overlap with zero are marked with a red box.

HVLT, Hopkins Verbal Learning Test; MoCA, Montreal Cognitive Assessment; PD, Parkinson's disease

6. Frequentist analysis

6.1. Group comparisons

Supplementary Table 7: Group comparisons of basal forebrain and hippocampus volume (normalized with respect to total intracranial volume) by ANCOVAs, including covariates for age, sex, and years of education. P-values from post-hoc tests are Bonferroni corrected for multiple comparisons.

	Posterior basal forebrain volume	Anterior basal forebrain volume	Hippocampus volume
ANCOVA model	$F_{2,1153}$ =23.1, p<0.001	$F_{2,1153}$=11.8, p<0.001	$F_{2,1153}$=4.0, p=0.001
HC vs asympt. GBA1	**p=0.006**	**p<0.001**	p=1.0
HC vs asympt. LRRK	**p=0.01**	**p<0.001**	p=1.0
Asympt. GBA1 vs asympt. LRRK2	p=1.0	p=1.0	p=1.0
iPD vs PD-GBA1	p=1.0	p=1.0	p=1.0
iPD vs PD-LRRK2	**p<0.001**	**p<0.001**	p=0.08
PD-GBA1 vs PD-LRRK2	**p=0.004**	p=0.07	p=1.0
HC vs iPD	**p<0.001**	p=1.0	p=0.10
HC vs PD-GBA1	p=1.0	p=1.0	p=1.0
HC vs PD-LRRK2	p=0.06	**p<0.001**	p=1.0
Asympt. GBA1 vs PD-GBA1	**p<0.001**	p=0.13	p=1.0
Asympt. LRRK2 vs PD-LRRK2	p=1.0	p=1.0	p=1.0

HC, healthy controls; iPD, idiopathic Parkinson's disease

6.2. Longitudinal associations with cognition

Supplementary Table 8: Beta coefficients for the effect of time in linear mixed models including cognitive scores as dependent variable, fixed effects for time, age, sex, years of education (and disease duration for PD groups), and random effects for intercepts and time. Models that indicated a significant (p<0.05) negative effect of time (i.e. cognitive decline) are marked in bold.
HVLT, Hopkins Verbal Learning Test; MoCA, Montreal Cognitive Assessment; PD, Parkinson's disease

	Idiopathic PD	PD-GBA1	PD-LRRK2	controls	asympt. GBA1	asympt. LRRK2
MoCA	**β=-0.07, p=0.002**	**β=-0.13, p=0.021**	β=0.03, p=0.34	**β=-0.06, p=0.034**	β=0.08, p=0.003	β=0.11, p=0.00014
Judgement of line orientation	**β=-0.05, p=0.003**	β=-0.11, p=0.18	β=0.03, p=0.38	β=-0.04, p=0.11	β=0.04, p=0.14	β=0.01, p=0.78
Letter number sequencing	**β=-0.09, p<0.001**	**β=-0.22, p=0.0009**	β=-0.03, p=0.37	β=-0.01, p=0.68	β=0.04, p=0.38	β=0.09, p=0.05
Symbol digit modalities test	**β=-0.10, p<0.001**	**β=-0.14, p=0.001**	**β=-0.08, p=0.007**	β=0.004, p=0.86	β=0.10, p=0.00003	β=0.05, p=0.02
Semantic fluency animals	β=-0.004, p=0.84	**β=-0.17, p=0.0004**	β=-0.06, p=0.053	β=-0.03, p=0.17	β=-0.04, p=0.17	β=-0.01, p=0.72
HVLT – delayed recall	β=-0.02, p=0.223	**β=-0.22, p=0.0002**	β=-0.02, p=0.64	β=0.03, p=0.26	**β=-0.11, p=0.00022**	**β=-0.08, p=0.03**

Supplementary Table 9: Beta coefficients for the interaction of time and posterior basal forebrain volume in linear mixed models including cognitive scores as dependent variable, fixed effects for posterior basal forebrain volume, time, age, sex, years of education (and disease duration for PD groups), the interaction between time and posterior basal forebrain volume, and random effects for intercepts and time. Models that indicated a significant ($p<0.05$) positive association between baseline volume and longitudinal cognition (i.e. smaller volumes associated with more severe decline in cognition) are marked in bold.
HVLT, Hopkins Verbal Learning Test; MoCA, Montreal Cognitive Assessment; PD, Parkinson's disease

	Idiopathic PD	PD-GBA1	PD-LRRK2	controls	asympt. GBA1	asympt. LRRK2
MoCA	**β=0.09, p=0.00004**	**β=0.12, p=0.033**	**0.07, p=0.03**	β=0.003, p=0.91	β=-0.05, p=0.048	β=-0.03, p=0.38
Judgement of line orientation	β=0.03, p=0.11	**β=0.19, p=0.020**	β=0.03, p=0.36	β=-0.01, p=0.72	β=0.02, p=0.76	β=0.01, p=0.83
Letter number sequencing	**β=0.07, p=0.00005**	**β=0.13, p=0.020**	β=0.06, p=0.08	β=-0.02, p=0.44	β=0.01, p=0.88	β=0.03, p=0.44
Symbol digit modalities test	**β=0.05, p=0.00382**	**β=0.11, p=0.007**	β=-0.01, p=0.71	β=-0.01, p=0.74	β=0.03, p=0.22	β=0.02, p=0.24
Semantic fluency animals	**β=0.04, p=0.016**	β=0.05, p=0.33	β=0.04, p=0.20	β=-0.01, p=0.66	β=0.05, p=0.06	**β=0.05, p=0.03**
HVLT – delayed recall	**β=0.06, p=0.0004**	β=0.01, p=0.84	β=0.05, p=0.13	β=-0.02, p=0.84	**β=0.08, p=0.005**	β=-0.04, p=0.23

Supplementary Table 10: Beta coefficients for the interaction of time and anterior basal forebrain volume in linear mixed models including cognitive scores as dependent variable, fixed effects for anterior basal forebrain volume, time, age, sex, years of education (and disease duration for PD groups), the interaction between time and anterior basal forebrain volume, and random effects for intercepts and time. Models that indicated a significant ($p<0.05$) positive association between baseline volume and longitudinal cognition (i.e. smaller volumes associated with more severe decline in cognition) are marked in bold.
HVLT, Hopkins Verbal Learning Test; MoCA, Montreal Cognitive Assessment; PD, Parkinson's disease

	Idiopathic PD	PD-GBA1	PD-LRRK2	controls	asympt. GBA1	asympt. LRRK2
MoCA	**β=0.07, p=0.0003**	β=0.03, p=0.54	**β=0.08, p=0.01**	β=-0.02, p=0.52	β=-0.05, p=0.1	β=-0.003, p=0.92
Judgement of line orientation	β=0.01, p=0.34	β=0.10, p=0.20	β=-0.03, p=0.35	β=-0.008, p=0.75	β=-0.04, p=0.47	β=0.003, p=0.94
Letter number sequencing	**β=0.07, p=0.0003**	β=0.08, p=0.19	β=0.02, p=0.48	β=-0.02, p=0.33	β=0.04, p=0.39	β=0.05, p=0.29
Symbol digit modalities test	**β=0.04, p=0.03**	β=0.03, p=0.53	β=-0.02, p=0.42	β=0.004, p=0.84	β=0.005, p=0.85	β=0.03, p=0.90
Semantic fluency animals	β=0.02, p=0.22	β=0.05, p=0.32	β=0.06, p=0.06	β=-0.01, p=0.66	β=0.008, p=0.77	**β=0.06, p=0.01**
HVLT – delayed recall	**β=0.06, p=0.001**	β=0.04, p=0.51	β=0.04, p=0.26	β=-0.005, p=0.85	β=0.03, p=0.27	β=-0.02, p=0.52
